# Cortico-Striatal Cross-Frequency Coupling and Gamma Genesis Disruptions in Huntington’s Disease Mouse and Computational Models

**DOI:** 10.1523/ENEURO.0210-18.2018

**Published:** 2018-12-21

**Authors:** Sebastien Naze, James Humble, Pengsheng Zheng, Scott Barton, Claudia Rangel-Barajas, George V. Rebec, James R. Kozloski

**Affiliations:** 1 T.J. Watson IBM Research Center, Yorktown Heights, NY 10598; 2 Department of Psychological and Brain Sciences, Indiana University, Bloomington, IN 47405

**Keywords:** fast-spiking interneuron, gamma, Huntington’s disease

## Abstract

Abnormal gamma band power across cortex and striatum is an important phenotype of Huntington’s disease (HD) in both patients and animal models, but neither the origin nor the functional relevance of this phenotype is well understood. Here, we analyzed local field potential (LFP) activity in freely behaving, symptomatic R6/2 and Q175 mouse models and corresponding wild-type (WT) controls. We focused on periods of quiet rest, which show strong γ activity in HD mice. Simultaneous recording from motor cortex and its target area in dorsal striatum in the R6/2 model revealed exaggerated functional coupling over that observed in WT between the phase of delta frequencies (1–4 Hz) in cortex and striatum and striatal amplitude modulation of low γ frequencies (25–55 Hz; i.e., phase-amplitude coupling, PAC), but no evidence that abnormal cortical activity alone can account for the increase in striatal γ power. Both HD mouse models had stronger coupling of γ amplitude to δ phase and more unimodal phase distributions than their WT counterparts. To assess the possible role of striatal fast-spiking interneurons (FSIs) in these phenomena, we developed a computational model based on additional striatal recordings from Q175 mice. Changes in peak γ frequency and power ratio were readily reproduced by our computational model, accounting for several experimental findings reported in the literature. Our results suggest that HD is characterized by both a reorganization of cortico-striatal drive and specific population changes related to intrastriatal synaptic coupling.

## Significance Statement

In Huntington’s disease (HD), functional impairments first impact movement (chorea, dyskinesia) and cognition (executive functions, abstract thinking). Neuronal dysfunction associated with deficits appear first in cerebral cortex and striatum, which displays abnormally strong power in the low γ band (25–55 Hz). Fast-spiking interneurons (FSIs) are a neuronal subtype putatively capable of forming networks that resonate at the γ frequency. Here, we show that FSI networks can implement an early routing bias for more abundant neuronal subtypes in striatum. Specifically, by analyzing *in vivo* electrophysiological recordings from HD mice at rest when γ is strong and using computational modeling, we identify coupling changes between cortex and striatum sufficient to generate periods of abnormal FSI network γ in response to slow wave cortical inputs.

## Introduction

The inherited neurodegenerative condition Huntington’s disease (HD) is caused by an excessive number of CAG repeats in exon 1 of the mutant huntingtin gene (mHTT; Ross et al., 2014). This gene is a critical player in broad and important intracellular functions such as endocytosis and trafficking (Velier et al., 1998; Nath et al., 2015). HD affects ∼0.013% of the population of Western countries (Bates et al., 2015), and age of onset varies, with a mean of ∼40 years. Symptoms appear slowly, include progressively worsening cognitive deficits and motor disturbances (chorea), and result in a fatal last stage involving severe and debilitating behavioral dysfunction (Roos, 2010). Postmortem histology and imaging studies revealed substantial cortical thinning owing mainly to a loss of cortical output neurons, and a drastic reduction in striatal size and weight (Cepeda et al., 2007).

Although no single animal model fully captures HD symptomatology and progression, two transgenic mouse lines reflect the wide phenotypic range and are currently used to assess mHTT’s mechanisms of action and test therapeutic strategies (Leuchter et al., 2017; for review, see Rangel‐Barajas and Rebec, 2018). The R6/2 mouse line, which contains exon 1 of mHTT, has been most widely investigated. Symptoms in this model show early onset (approximately two months of age) and progress over the next few months to immobility and death (Mangiarini et al., 1996; Li et al., 2005). The Q175 mouse line is a knock-in model that expresses mHTT in its proper genetic context and shows symptoms with onset in adulthood (more than five months of age) that progress rapidly in homozygous (HOM) but more slowly in heterozygous (HET) animals (Menalled et al., 2012).

Electrophysiologically, both R6/2 and homozygous Q175 mice show increased low-frequency γ power (25–50 Hz) in dorsal striatum (Hong et al., 2012; Rothe et al., 2015), which when coherent with cortical oscillations, has been implicated in motor decisions and cognitive learning (Berke et al., 2004; Pennartz et al., 2009; Popescu et al., 2009). As the primary input structure of the basal ganglia, the striatum receives topographically ordered afferent connections from all areas of cortex (Hintiryan et al., 2016). Although the role of the striatum in integrating cortical states for downstream behavioral processing through multiple parallel pathways has been heavily investigated (Steiner and Tseng, 2016; Jaeger and Kita, 2011; Kreitzer and Berke, 2011), the functional implications of striatal γ are still poorly understood. Some evidence suggests that γ emerges from fast-spiking interneurons (FSIs) within the striatal microcircuit and supports fast action-selection by biasing the circuit from adversarial to automatic behavior selection (Gage et al., 2010; Berke, 2011).

In addition, other frequency bands in brain areas that interact with striatum are also disrupted in HD. For example, δ band (0.5–4 Hz) and α band (8–12 Hz) EEG powers are increased in cortex of HD patients, and the relative anteroposterior gradient is lost (Hunter et al., 2010). Here, we investigate the relationships between different frequency bands and the circuit disruptions that might explain them. By analyzing data from experimental mouse models and simulating a neuronal network of FSIs in a computational model, our aim was to inform striatal circuit modifications in HD that might also explain changes in the behavioral phenotype.

We hypothesized that disruptions giving rise to abnormal power in striatal frequency bands emerge from one or more of the following changes: (1) abnormal cortical activity, (2) abnormal cortico-striatal integration and/or coupling, and (3) disruptions to intrastriatal circuitry. We aimed to test each hypothesis by first analyzing phase relationships between and within channels recorded simultaneously from electrode bundles chronically implanted in both motor cortex and dorsal striatum of R6/2 mice and dorsal striatum of hetero- and homozygous Q175 mice. We examined phase-amplitude coupling (PAC) between cortical δ and striatal γ oscillations using a signal processing routine for extracting transient increases in γ band power (i.e., “γ events”). We then created a computational network model of FSIs inspired by the work of Traub et al. (2001) and subjected it to the same analysis as our experimental data to investigate striatal γ. We used experimental δ band cortical local field potential (LFP) time series, shown to be unchanged between HD and wild type (WT), as inputs to our striatal FSI network and constrained by connectivity statistics and synaptic time constants from current literature on mouse striatal FSIs (Cepeda et al., 2013). Either WT or HD δ band cortical driving inputs resulted in HD γ statistics by varying gap junctional coupling between FSIs, intra-FSI synaptic conductances, or cortico-FSI synaptic strength.

## Materials and Methods

### Experimental procedures

Data were obtained from two HD models and their respective WT background line littermate controls, each obtained from The Jackson Laboratory. R6/2 mice (B6CBA-TgN [HD exon 1] 62Gpb), a truncated HD model, express an expanded CAG repeat in exon 1 of the human HD gene. In Q175 mice (C57Bl/6), a knock-in HD model, the expanded CAG repeat is “knocked into” its proper genetic context. Only male mice were used. All mice were genotyped to confirm CAG repeat length as determined by PCR from tail tissue samples as previously described (Miller et al., 2008). For both models, CAG repeat lengths ranged between 125 and 185. Both models were symptomatic at the time of recording: 8–10 weeks of age for R6/2s (Mangiarini et al., 1996) and 30–45 weeks of age for Q175s (Menalled et al., 2012). Animals were housed under controlled temperature and humidity conditions in an AAALAC-approved facility in the Psychology Building at Indiana University, Bloomington. Mice were maintained on a 12/12 h light/dark cycle with lights on at 07:30 A.M. and with free access to food and water. Testing occurred around the mid-point of the light phase. Animal use was in accord with the National Institutes of Health Guide for the Care and Use of Laboratory Animals and approved by the local Institutional Animal Care and Use Committee. All efforts were made to minimize suffering and the number of animals used in these experiments.

Details of the experimental procedures have appeared elsewhere (Hong et al., 2012). For R6/2 experiments, each electrode bundle consisted of four recording micro-wires (each 25-μm diameter insulated stainless steel) and one ground wire (50-μm diameter uninsulated stainless steel). Each wire was friction-fitted to gold-plated pin connectors in polyphenylene sulfide insulators. Two sets of insulators were glued together to record simultaneously from primary motor cortex (M1) and dorsal striatum; one micro-wire bundle was cut to 0.5 mm in length, while the second was cut to 3.0 mm. For Q175 experiments, one electrode bundle with eight recording micro-wires and one ground wire targeted dorsal striatum. Cortical channels are referenced to cortical reference electrode and striatal channels are referenced to striatal reference electrode (both reference electrodes are connected to the recording system’s amplifier ground). In all cases, the head-mounted assembly was designed to be lightweight and well tolerated by the mice (Miller et al., 2008; Hong et al., 2012).

For electrode implantation, mice received meloxicam (1 mg/kg; s.c.) followed by anesthesia with a mixture of chloral hydrate and sodium pentobarbital (chloropent: 170 mg/kg chloral hydrate and 40 mg/kg sodium pentobarbital) administered intraperitoneally at 0.4 ml/100 g body weight. Mice were secured in a stereotaxic frame, and following a midline scalp incision, a hole was drilled +0.5 mm anterior and ±1.5 mm lateral to bregma (Paxinos and Franklin, 2001). Two additional holes in the contralateral hemisphere were used for placement of stainless-steel anchor screws. Electrode bundles were lowered into M1 and/or dorsal striatum (0.5 and 3.0 mm ventral to brain surface, respectively). Dental acrylic fixed the electrode assembly in place on the skull. Mice were allowed one week of recovery before testing, which continued at regular intervals (weekly for the R6/2 experiments and monthly for the Q175 recordings). On LFP recording days, male gold pins attached to a lightweight flexible wire harness equipped with field-effect transistors were inserted into the head-mounted electrode assembly. The harness was attached to a swivel to allow free movement. LFPs were routed through preamplifiers with 1000× gain and 0.7–170 Hz filters (Plexon). Mice were placed in an open-field arena (25 × 18 cm with outwardly angled walls 17 cm high) housed in a sound-attenuating and electrically shielded recording chamber. After a 5- to 10-min habituation period, data were collected for 20 min. Open-field behavior was videotaped and synchronized with electrophysiological recording. Behavioral episodes were time-stamped and categorized as quiet rest, grooming, or exploration based on visual observation of episodes sustained for at least 3 s as previously reported (Hong et al., 2012). Videotapes were coded by independent observers blind to genotype.

### Data analysis

**Figure 1.**
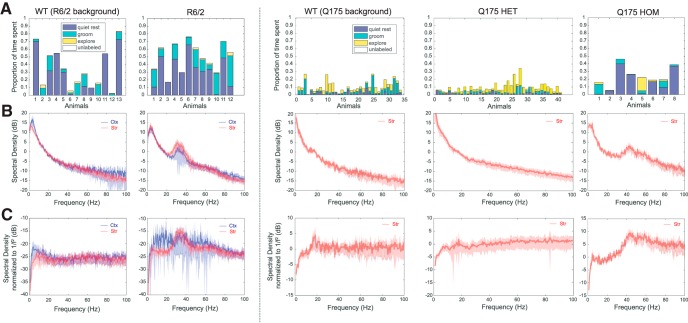
Proportion of time spent in each labeled state and corresponding relative power spectra during quiet rest for each animal type. ***A***. Proportion of time spent in each of the three labeled states, normalized by total recording time. Behavioral states needed to be sustained for at least 3 seconds to be labeled. ***B***. Power Spectrum Density (PSD) of cortical (blue) and striatal (red) recordings during quiet rest, averaged over all animals (SD shown by shaded region). Each channel’s PSD was normalized by its total power over the beta (15-20 Hz) frequency band so that the average spectrum was not biased toward higher amplitude channels. ***C***. Same as ***B*** with each channel normalized by 1/f^2^.

All datasets were archival when shared with researchers from IBM, and no new experiments were suggested, designed, or performed based on these analyses. The first dataset contained simultaneous recordings for 12 R6/2 mice and 13 corresponding WT mice. All mice in this dataset had electrodes implanted in dorsal striatum and motor cortex. The behavioral states of the animals were time-stamped as “quiet rest,” “grooming,” or “exploration,” based on visual observation of the sustained state for at least 3 s. Figure 1*A* shows the proportion of time in each state for each animal of a specific type, and time not labeled according to any of the three behavioral states caused proportions to sum to less than one for each animal.

The second dataset contains 8 homozygous Q175 mice, 34 heterozygous Q175 mice and 41 corresponding WT mice. Fewer periods of quiet rest for the heterozygous Q175s and WTs were compensated by the larger number of animals than those in the first dataset (Fig. 1*A*).

### Amplitude, frequency, and phase modulations

**Figure 2.**
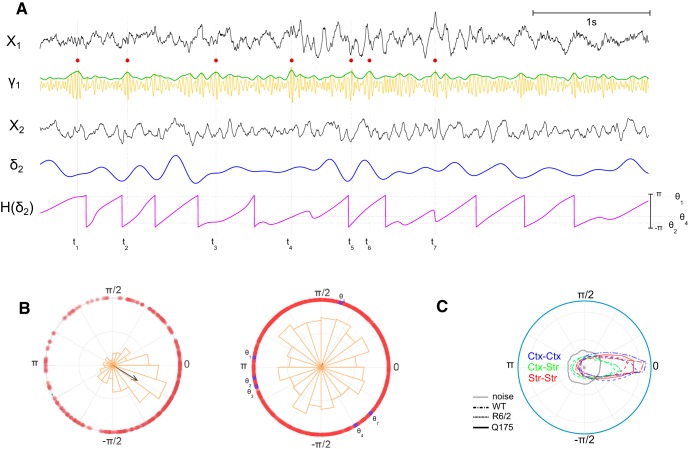
Time series analysis and delta band phase difference during quiet rest. ***A***. Time series analysis. Raw LFP simultaneous recordings from 2 electrodes (X_1_, X_2_) are processed separately. X_1_ is band-pass filtered at 25-50 Hz to extract low-gamma signal γ_1_, and gamma events (red circles) are extracted from the envelope of this gamma signal (green trace). X_2_ is low-pass filtered at 4 Hz cutoff to extract the delta band activity (δ_2_), of which the instantaneous phase is extracted using the angle of the Hilbert transform (H(δ_2_)). The phase thetai at which gamma events occur on the delta oscillation is derived from the value of H(δ_2_) at the time of the event ti. Traces taken from WT animal #1 for illustrative purpose. ***B***. Example of projections onto the unit circle of all delta phases at which gamma events occur, for one pair of channels. Each red circle indicates the phase of the event. The distribution of phases at which gamma events occur is shown in a polar histogram (orange). The arrow pointing from the origin represents vector strength of phase-locking (length) and mean phase of gamma events (orientation). Left: channel with strong gamma event delta phase-locking. Right: channel without gamma event delta phase-locking. Sample phases from time series in A) are denoted in purple. ***C***. Circular distributions of delta phase difference across channels located in cortex only (Ctx-Ctx, blue), in cortex and striatum (Ctx-Str, green) and in striatum only (Str-Str, red). Animal types are denoted by different line types (legend inset). Delta phase difference between two white noise signals is shown in gray. 0° stands for zero phase difference i.e. synchronization of delta rhythm oscillations across channels.

Phase and amplitude modulations were computed based on the analytic phase and analytic amplitude derived from the Hilbert transform (Freeman, 2004a,b, 2007) for the δ band activity. The LFP signal was adjusted to have zero mean then low pass filtered with 4 Hz cutoff frequency (Fig. 2*A*). We used a 4th order Butterworth zero-phase forward and reverse filter to avoid phase distortion. The derived δ band signal ($x^{\delta }$) was Hilbert-transformed (H) resulting in a representation of the signal in the complex plane from which we could extract the analytic amplitude and phase. (Note that δ does not symbolize the Kronecker δ, a commonly used input-response function in signal processing, but instead the δ band of the signal of interest.)

The analytic amplitude A_j_ of electrode *j* is computed as the square root of the sum of squared real and imaginary parts of the Hilbert transform:

$$A_{j}\left(t\right)=\sqrt{{Re\left(H\left(x^{\delta }\left(t\right)\right)\right)}^{2}+{Im\left(H\left(x^{\delta }\left(t\right)\right)\right)}^{2}}$$

**Figure 3.**
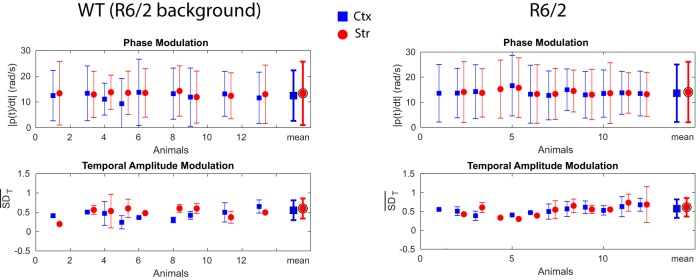
Delta phase and amplitude modulations are comparable across cortex and striatum. Top: Phase modulation computed as the slope of the unwrapped analytic phase in the delta band, averaged over cortical (blue squares) and striatal (red circles) channels for each animal. Larger symbols on the right indicate the mean slope, corresponding to the mean frequency of the filtered signal (note that 1Hz = 2π rad/s). The degree of phase modulation resides in deviations from the mean, indicated by error bars. Bottom: Amplitude modulation (AM) computed as the standard deviation (SD) of the squared analytic amplitude in the delta band for each cortical (blue squares) and striatal (red circles) channels. Larger symbols on the right indicate the averaged SD across cortical channels per animal (see Methods), a value close to 0 indicates weak AM and deviation from 0 indicates stronger AM signals. Note that some animals do not have channels in every structure (no symbols), or have only one channel per structure (zero SD).

For each animal, we normalized the squared analytic amplitude of each channel by the averaged squared analytic amplitude over channels: ‖Aj2(t)‖=Aj2(t)∑jAj2(t). The measure of amplitude modulation in time (over the whole quiet rest period) for one channel is then given by the SD of the normalized squared analytic amplitude ${SD}_{T}(j)=SD\left(\left\|{A_{j}}^{2}\left(t\right)\right\|\right)$, and we used the average of those SD s across channels as a global measure of temporal amplitude modulation for each animal. (The error bars in Fig. 3 are then the SD of ${SD}_{T}$ across channels.)

The analytic phase $P_{j}$ of electrode *j* is computed as the arctangent of the ratio of the imaginary over the real part of the Hilbert transform:

$$P_{j}\left(t\right)=\tan^{-1}\left(\frac{Im\left(H\left({x_{j}}^{\delta }\left(t\right)\right)\right)}{Re\left(H\left({x_{j}}^{\delta }\left(t\right)\right)\right)}\right)$$

The arctangent was calculated using the four-quadrant inverse tangent function (atan2 in MATLAB), which takes values between -π and π and, when reaching π, falls to -π once in each cycle (Freeman, 2007). The resulting time series $P_{j}(t)$ is not continuous (a saw tooth curve). to evaluate the phase slip of a single analytic phase time series over large recording periods, the disjoint phase sequences were first straightened by adding 2π to the arctangent function at each jump to get the unwrapped analytic phase $p_{j}(t)$. The slope of the ramp, computed by the instantaneous derivative $D_{j}(t)$, then gave the mean frequency over the duration of the time interval when fitted to a straight line.

$$D_{j}\left(t\right)=|p_{j}\left(t\right)|-|p_{j}\left(t-1\right)|$$

The deviation from this mean frequency indicates a phase slip, such that a higher SD of $D_{j}$ across the time series indicates stronger phase modulation in channel *j*. The mean frequency averaged across cortical channels for each animal is shown in Figure 3 and the averaged phase modulation across channels is indicated by error bars.

The phase difference at time *t* between any pair of electrodes is calculated by subtracting the instantaneous phase $P_{j}(t)$ of signal $x_{j}(t)$ by the instantaneous phase $P_{i}(t)$ of signal $x_{i}(t)$:

$$P_{i,j}\left(t\right)=P_{j}\left(t\right)-P_{i}\left(t\right)$$

### Extraction of γ events

Analysis was restricted to periods of quiet rest (Hong et al., 2012), the most frequently observed behavioral state among WT, R6/2, and Q175 HOM mice (Fig. 1*A*), during which significant increases in γ power reliably occurred. These recordings X were zero-centered by subtracting the mean signal $〈X〉$ and bandpass filtered (BPF) within the low γ frequency range (25–55 Hz), then full-wave rectified.

$$x^{\gamma }(t)=\left\|{BPF}_{25<f<55}(X-〈X〉)\right\|$$

The resulting signal was low-pass filtered (LPF) at 15 Hz cutoff to yield the envelop of the γ band signal $x^{E(\gamma )}\left(t\right)$:

$$x^{E(\gamma )}\left(t\right)={LPF}_{f<15}(|x^{\gamma }\left(t\right)|).$$

Both steps used a 4th order Butterworth filter with zero-phase forward and reverse filtering to avoid phase distortion.

Finally, the envelope signal $x^{E}$ was *z*-score normalized: $x_{z}^{E}\left(t\right)=\frac{x^{E}-〈x^{E}〉}{sd(x^{E})}$ where *sd(x^E^)* is the standard deviation of variable *x^E^*.

Epochs during which the normalized signal exceeded a threshold (2.5 SD from the mean) were considered γ events, the peaks of which were among the highest 0.6% of detected peaks and marked the exact timing of each strong fluctuation in LFP γ power:

$$\Upsilon_{event}\left(t_{peak}\right)={\max\left(X_{z}^{E}(T)\right)}_{T\in t},X_{z}^{E}(T)\in {\left(x_{z}^{E}(t)>2.5\right)}_{T\in t}$$

where T represent the set of time points at which $x_{z}^{E}(t)$ exceeds 2.5, and $X_{z}^{E}(T)$ is the value of the z-scored γ envelope at those points. It is important to note that the γ event extraction algorithm depends on a statistical threshold of the given time series, and therefore detects events regardless of the overall signal power in the γ band. An exclusion criterion was applied to exclude events resulting from broad band power increases by selecting only γ events that do not co-occur with transient power increase in α (8–12 Hz), β (13–24 Hz), and high γ (>60 Hz) bands. In the case of a flat power spectrum with weak power in the γ range (Fig. 1*B*,*C*, WTs), the extracted γ events may only represent noise fluctuations, but when γ power is stronger (Fig. 1*B*,*C*, R6/2 and Q175 HOM), the events additionally capture transient and significant (with respect to other frequency bands) increases in γ power. There were no qualitative differences by visual inspection between γ events across the different mouse strains.

### δ Phases at γ events

We determined the distribution of δ phases at which γ events occur using the exact time of a γ event, $\theta_{x^{\delta }}\left(t_{peak}\right)$ and the corresponding phase derived from the value of the imaginary part of the Hilbert-transformed δ signal (Fig. 2*A*). The resulting distribution of γ event phases describes how γ events phase-locked to the slower δ oscillation. A circular normal (von Mises) distribution indicates phase-locking with strength proportional to its kurtosis (Fig. 2*B*, left), whereas a uniform distribution denotes the absence of phase-locking (Fig. 2*B*, right). Statistical significance of non-uniformity of the phase-locking distribution was assessed using a Rayleigh test. Statistical tests for unimodality versus multimodality of the phase distribution was performed using Hartigan’s Dip test (Hartigan and Hartigan, 1985), of which the *p* value p→1 when the distribution is unimodal and p→0 ∧ dip≈0.1 when the distribution is multimodal. Channels with <50 γ events, due to quiet rest periods being too short, were dismissed from the analysis. Circular statistics were performed using the CircStat MATLAB toolbox (Berens, 2009).

### Correlation between γ events

The temporal correlation of γ events was computed across pairs of recordings. For this correlation analysis, γ events previously represented as single time points corresponding to amplitude maxima in the γ band-passed envelope were transformed into a binary time series by computing the *z* score and thresholding the envelope at 2.5 times the SD:

Υevent(T)={1    XzE(T)>00    otherwise.

Therefore, temporal correlations of γ events between channels were computed from continuous periods of elevated γ power in contrast to the single timestep $t_{peak}$ described in the previous section. Pearson correlation coefficients between pairs of electrodes’ binary time series were computed using MATLAB’s built-in corr function.

### Spectral analysis

Power spectral densities were computed using Thomson’s multi-taper power spectra estimate in MATLAB. Briefly, the multi-taper method uses multiple windows (tapers) chosen based on their statistical independence to estimate spectral density. In contrast, the standard Welch method uses averaged periodograms constructed from multiple overlapping segments of the signal to reduce variance of the spectral density estimate. This introduces redundancy and is more computationally intensive. Fourier coefficients were computed between 0.5 and 100 Hz at intervals of ∼0.5. Power line artifact at 60 Hz was removed by choosing an increment close to but not equal to 0.5, such that the amplitude of the Fourier coefficient at exactly 60 Hz was not computed. Finally, coefficients were normalized (unless otherwise specified) to the average amplitude of the β frequency band ($f_{\beta }$ = [15–20] Hz; Fig. 1*B*), which is not modulated in animals at rest, thereby removing any bias resulting from constant energy over the whole frequency spectrum (see Discussion). Spectra with a $1/f^{2}$ normalization are shown in Figure 1*C* to emphasize the peak in the low γ frequency band.

Time-frequency transformations (i.e., spectrograms) were performed by convolving the signal s(t) with a Morlet wavelet w(t) as described in Tallon-Baudry et al. (1996) and von Nicolai et al. (2014):

Ψ(t,f)=s(t)⊗w(t,f).

The complex Morlet wavelet has a Gaussian shape both in the time (SD $\sigma_{t}$) and frequency (SD $\sigma_{f}$) domains around its central frequency $f_{0}$:

$$w\left(t,f_{0}\right)=A\cdot e^{\;j2\pi f_{0}t}\cdot e^{-t^{2}/2{\sigma_{t}}^{2}}$$

where $A=\frac{\sqrt{2\pi }}{F_{s}\sigma_{t}}$ is a normalization factor, $\sigma_{t}=\frac{1}{2\pi \sigma_{f}}$, σf=f0q, and q is a constant spectral width of five cycles. We also used a constant temporal width of $4\sigma_{t}$ and set $f_{0}$ to span values from 0.5 to 100 Hz by steps of 0.5 Hz. We kept only the wavelet coefficient by removing the imaginary part of the output:

$$\Psi_{c}\left(t,f\right)=R(\Psi \left(t,f\right)).$$

### Complex coherence

We computed the complex coherence based on the cross spectral density estimates $S_{i,j}\left(f\right)$between channel *i* and channel *j* for the frequency band corresponding to low-γ: $f\in \left[25,55\right]Hz$

$$S_{i,j}\left(f\right)\equiv 〈X_{i}\left(f\right)X_{j}^{\;*}\left(f\right)〉$$

where $X_{i}\left(f\right)$ and $X_{j}(f)$ are the discrete Fourier transforms of time series of channel *i* and *j*, respectively, * means complex conjugation and 〈〉 denotes mean expectation value. The coherence is then defined as the cross spectrum normalized by the square root of the products of auto-spectra:

$$C_{i,j}\left(f\right)\equiv \frac{S_{i,\;j}(f)}{\sqrt{S_{i,\;i}\left(f\right)S_{i,\;j}\left(f\right)}}$$

and is complex valued for each frequency value *f* if channels *i* and channel *j* are phase shifted at this frequency. Expectation values of spectral densities were estimated during periods of quiet rest when a γ event occurred in at least one of the two channels.The projection of the complex valued coherence onto the unit circle in polar coordinates is done using Euler’s identity, where the norm *r* is the magnitude of the coherence (1 when signals have exactly the same frequency content) and angle φ is the average phase shift (0 if signals are perfectly in phase at frequency *f*). Re-writing $C_{i,j}=a+ib$ with $a=Re(〈C_{i,j}〉)$ and $b=Im(〈C_{i,j}〉)$, then

$$r=\sqrt{a^{2}+b^{2}}$$

φ=tan-1⁡ba

### Modeling

We aimed to create a model capable of reproducing electrophysiological properties such as γ band rhythmogenesis and inter-frequency relationships between γ and δ oscillations. These broad validation targets seemed accessible to several modeling approaches, each of which were useful for investigating interactions between neural entities likely necessary to yield these phenomena. Models ranging from biophysically realistic neuron models (e.g., the Hodgkin–Huxley formalism) to mean-field approximations (e.g., the Wilson–Cowan model) were considered, and each presented pros and cons (Deco et al., 2008). We chose a model of intermediate complexity, the integrate-and-fire (IAF) neuron model of Mihalaş and Niebur (2009), which is well established and known for its flexibility in reproducing a set of well-defined firing patterns. In addition, this model is formalized in a computationally efficient implementation, allowing for efficient and very large network simulations. Finally, our approach of using networks of point neurons to investigate dynamics and information transmission in oscillatory networks of spiking neurons has been used successfully to explain several complex mechanisms, from neuronal synchronization (Brunel, 2000) to sensory coding (Mazzoni et al., 2008).

### Neuron model

We used a generalized IAF model, which previously reproduced a wide range of spiking dynamics with a small number of parameters (Mihalaş and Niebur, 2009). The normal form of the equations for ionic currents *I_j_*, membrane potential *V* and instantaneous firing threshold Θ of a single unit are

$$\frac{dI_{j}}{dt}=-kI_{j}(t);\;j=1,\ldots ,N$$

$$\frac{dV}{dt}=\frac{1}{C}\left(I_{e}+\sum_{j}I_{j}\left(t\right)+\sum_{i}I_{s_{i}}(t)+I_{gap}(t)-G(V\left(t\right)-E_{L})\right)$$

$$\frac{d\Theta }{dt}=a\left(V\left(t\right)-E_{L}\right)-b(\Theta \left(t\right)-\Theta_{\infty })$$

for $V\left(t\right)<\Theta_{r}$. When $V\left(t\right)\geq \Theta_{r}$:

$$I_{j}\left(t\right)\leftarrow R_{j}+I_{j}\left(t\right)$$

$$V\left(t\right)\leftarrow V_{r}$$

$$\Theta \left(t\right)\leftarrow {max(\Theta \left(t\right),\Theta }_{r}),$$

Where the subscript r denotes the reset value of the variable after $V\left(t\right)\geq \Theta_{r}$ and a spike is elicited, while subscript *j* denotes the ionic current (here 2, as in the original model). The membrane and threshold reset values were $V_{r}=-0.07V$ and $\Theta_{r}=-0.06V$. $I_{s}$ and $I_{gap}$ are synaptic and gap junctional currents injected from the network. In the original published model, voltage and threshold were reset on the time step subsequent to a spike. Our model holds voltage at threshold for an additional 1 ms of simulated time after a spike, to approximate the width of the physiologic action potential and allow time for current to flow through gap junctions. Coefficients *a* and *b* describe the dependence of the threshold on the membrane potential and were set to a = 5 s^−1^ and b = 10 s^−1^. Membrane capacitance was set to unity, C = 1, the leak rate of the membrane to G = 50 s^−1^, and the resting membrane potential (reversal potential of the leak current) to E_L_ = –0.07 V. The target threshold value was set to $\Theta_{\infty }=-0.05V$, the current update constants R_1_ = 0 and R_2_ = 1 as well as all other parameters were set according to values from the original published model (Mihalaş and Niebur, 2009) for Class 2 excitable neurons.

### Coupling

We modeled GABAergic synapses using α functions (Ermentrout and Terman, 2010) such that inhibitory postsynaptic currents were:

$$I_{s}(t)=g_{s}s_{f}(t)$$

$$\overset{˙}{s_{r}}=\frac{-s_{r}+\delta (t-t^{*})}{\tau_{r}}$$

$$\dot{s_{f}}=\frac{{(-s_{f}+s}_{r})}{\tau_{f}},$$

where t^*^ denotes the time of a pre-synaptic spike, $\tau_{r}=0.8ms$, and $\tau_{f}=12ms$, the rise and fall synaptic time constants (Taverna et al., 2008).

Gap junctional coupling was modeled as a linear difference term added to the membrane potential equation using a scalar factor ρ, which represents in normalized arbitrary units the conductance of neuron to neuron coupling. The current flowing into neuron *j* coupled to neuron *k* across their gap junctions is then:

$$I_{{gap}_{j}}\left(t\right)=\sum_{k\in K}{\rho (V}_{k}\left(t\right)-V_{j}(t))$$

where *K* is a set of neurons coupled to neuron *j* through gap junctions (bidirectionally, see network topology below).

### Network structure

FSIs were populated evenly on a 3D mesh of dimension 27 × 27 × 27, totaling 19,683 neurons. Neurons were separated from each other horizontally and vertically by ∼113 µm in the cubic mesh, such that their density (693/mm^3^) was within the range of experimentally observed striatal FSIs in mice (500–1250 neurons/mm^3^; Luk and Sadikot, 2001). Each neuron was connected via a gap junction to each neighbor within a radius of 120 µm with a probability of 0.35, and via a GABAergic synapse to each neighbor within a radius of 339 µm with a probability of 0.58 as described in Gittis et al. (2010).

### Cortical drive

A set of 27 “cortical units” arranged on a 3 × 3 × 3 mesh, project topographically to the FSI network without overlap, resulting in each cortical unit driving a set of 9 × 9 × 9 FSIs. This drive is implemented using experimental cortical LFP recordings, which were pre-processed to approximate the cortico-striatal excitation. Each cortical LFP was adjusted to have zero mean and then half-wave rectified, such that only positive signal deflections from the mean were injected as fluctuating excitatory drive into the FSI population. Before injection, the signal was low-pass filtered at 4-Hz-cutoff frequency to isolate the δ frequency band from the rest of the signal, *z*-score normalized as above, and scaled (by a factor referenced in the figures as “CtxFSI”) to elicit activity from the FSIs. The logic behind using the filtered experimental signal in the δ band as input to the FSI units is to reflect slow fluctuations in excitation originating from cortex. Thus, the cortico-striatal drive into the model FSI network represented a scaled normalized δ signal derived from real cortical LFPs.

### LFP reconstruction

Modeled LFPs were reconstructed by applying a 3D Gaussian kernel ($K_{LFP}$) to a specific region of the striatum centered on a recording point (electrode) with coordinates (*x_e_, y_e_, z_e_*) and computing the kernel-weighted conductance of extracellular medium within or nearby that region:

$$K_{LFP}\left(e,n\right)=\frac{1}{\sqrt{2\pi }\sigma }e^{-\frac{{\left(x_{n}-x_{e}\right)}^{2}+{\left(y_{n}-y_{e}\right)}^{2}+{\left(z_{n}-z_{e}\right)}^{2}}{2\sigma^{2}}}$$

where (*x_n_*, *y_n_*, *z_n_*) are the coordinates of unit *n* from the set of neurons *N*, located within a distance of ∼1.5mm (13 spatial units on the mesh) from the recording point, and σ is the SD of the spatially weighted field recorded by the electrode (we used σ = ∼300 µm or 2.65 mesh units). We used a 4 × 4 × 4 3D grid of 64 recording electrodes to reconstruct LFPs from across the striatal FSI network. Electrode grid points were spaced by ∼750 µm in each dimension, allowing some overlap between the sources from which recorded signals were reconstructed. Spatial scales of integration represented an upper bound (informed by Buzsáki et al., 2012; Einevoll et al., 2013) and were consistent with other theoretical point-neuron discrete reconstructions of the LFP (Mazzoni et al., 2015). We also computed the LFP including active membrane currents (rather than synaptic only) without qualitative difference using our model.

Our approach so far is simpler, but phenomenologically equivalent to the formulation of extracellular field potential reconstruction U used for Current Source Density Analysis of LFP and EEG (Nicholson and Freeman, 1975), in that it also assumes isotropic point-current sources:

$$U=\frac{1}{4\pi \sigma }\sum_{j}\frac{I_{j}}{r_{j}},$$

where $r_{j}$ is the distance between a current source $I_{j}$ and the electrode, and *σ* is the conductivity of the homogenous neural medium.

Finally, to account for the lack of specific spatial orientation of neuronal populations in the striatum (in contrast to cortical pyramidal cells for example), we also computed the modeled LFPs acknowledging for directions’ heterogeneity of the electrical fields generated by each neuronal unit (termed volumetric unit’s field). Each volumetric unit’s field was given a random orientation in 3D space by creating a point in spherical coordinates with zenith ρ∈[0,π[ and azimuth φ∈[0,2π[ drawn from a uniform distribution. The single neuron’s electric field was then represented by a unit vector pointing in this random direction, and its contribution to the nearby electrode was scaled both by the 3D Gaussian kernel described above and its orientation affinity to the electrode. The orientation affinity scalar $\xi_{n}$ of neuron n was derived from subtracting the distances from the electrode of the origin ($d_{o}$) and the tip ($d_{s}$) of the unit vector representing the volumetric units’ field emanating from the neuron.

$$\xi_{n}=d_{o}-d_{s}$$

where $d_{o}=\sqrt{{\left(x_{n}-x_{e}\right)}^{2}+{\left(y_{n}-y_{e}\right)}^{2}+{\left(z_{n}-z_{e}\right)}^{2}}$ and $d_{s}=\sqrt{{\left(x_{n}+\cos\rho_{n}\sin\varphi_{n}-x_{e}\right)}^{2}+{\left(y_{n}+\sin\rho_{n}\sin\varphi_{n}-y_{e}\right)}^{2}+{\left(z_{n}+\cos\rho_{n}-z_{e}\right)}^{2}}$

The LFP signal reconstructed for electrode *e* then reads:

$$V_{LFP}\left(e,t\right)=\sum_{n\in N}\xi_{n}K_{LFP}\left(e,n\right)I_{n}(t)$$

where $I_{n}$ are synaptic currents, $K_{LFP}$ is the extracellular conductance kernel, and $\xi_{n}$ is the orientation affinity of the neuron with respect to the electrode *e*.

### Code accessibility

The computational model was implemented in IBM Model Graph Simulator, which is the core parallel processing architecture for model description (using the Model Description Language, MDL) and resource allocation (using the Graph Specification Language, GSL) of the Neural Tissue Simulator (Kozloski and Wagner, 2011). The Model Graph Simulator software is experimental. Readers are therefore encouraged to contact the authors if interested in using the tool. A single simulation of ∼20,000 FSI units for 100 s took ∼2 h of computing time on a local Lenovo P910 desktop machine (2 Ghz, 64 bit, 14 cores, 2 threads/core, 64 Gb of RAM). Scripts written in MDL and GSL are hosted by *eNeuro*.

## Results

We analyzed phase and amplitude relations across δ and γ frequency bands among signals recorded simultaneously from multiple electrodes in motor cortex and dorsal striatum. Recordings occurred in HD models at ages when both were symptomatic (8–10 weeks for R6/2s) and (30–45 weeks for Q175s) and at comparable ages in corresponding WT background controls. We focused on periods of quiet rest because they are evident in sufficient numbers in both HD models and abnormal cortico-striatal γ bands had been identified in R6/2 mice at rest (Hong et al., 2012). We assessed phase differences across channels in the δ frequency band, and then the relationship between the distribution of transient increases in γ power (γ events) and the phase of the δ oscillation. γ Events occurred at a preferred phase of the δ oscillation, indicating functional coupling between the two frequency bands (Hyafil et al., 2015), and we found this coupling to be exaggerated in HD mouse models. We next assessed inter-γ event distributions and correlations between events across electrodes to provide insight into physiologic processes that may account for excess γ genesis in dorsal striatum. Finally, we report on results from simulations of a network model of striatal FSIs that reproduces many features of the analysis. We used the model to investigate varying cortico-striatal and striato-striatal interactions that are not experimentally accessible, examining their role in several possible origins of striatal γ. Our analysis focused on periods of quiet rest (Fig. 1*A*). Note that striatum refers to dorsal striatum for experimental analysis as it corresponds to electrodes location. The computational model is however agnostic of striatal physiologic heterogeneity, and therefore, we do not explicitly distinguish between striatal subfields.

### Cortical δ band statistics do not explain HD phenotype

#### δ Phase and amplitude modulations are comparable across cortex and striatum

We computed measures of amplitude and phase modulation based on the analytic amplitude and phase reconstructed from the Hilbert transform of the filtered cortical recordings in the δ band (see Materials and Methods and references therein). The results of these measurements are shown in Figure 3, where a measure of amplitude modulation is the SD of individual channels’ analytic amplitude normalized by their mean, and a measure of phase modulation is the SD of the instantaneous derivative (slope) of the unwrapped analytic phase. These statistics extracted from cortical channels in R6/2 animals were not different from their corresponding WT background, suggesting that cortical δ oscillations are not affected by the disease in animal models. Comparisons of the means of distributions of WT and R6/2 amplitude and phase modulations were performed using a two-sample Kolmogorov–Smirnov test (K-S test) and differences were rejected with respective *p* values (amplitude/phase) 0.49/0.94 for WT Ctx versus Str, 0.99/0.82 for R6/2 Ctx versus Str, 0.11/0.25 for WT Ctx versus R6/2 Ctx, and 0.41/0.65 for WT Str versus R6/2 Str. Comparisons were also performed for WT versus Q175 animals (results not illustrated), and K-S tests gave *p* values (amplitude/phase modulation) 0.42/0.29 for WT versus Q175 (heterozygous), 0.81/0.04 for WT versus Q175 (homozygous), and 0.73/0.63 for Q175’s homozygous versus heterozygous. This overall suggest no difference in amplitude modulation for δ band signals recorded in cortex and striatum, and little difference if any in phase modulation.

### δ Band synchrony is strong within and between cortex and striatum

Synchronization between δ band signals in motor cortex and dorsal striatum was assessed by comparing the phase differences between channels. The phase difference distribution shows a strong peak around zero (Fig. 2*C*), indicating zero-lag synchronization across all electrodes in the δ band, whether within motor cortex, within dorsal striatum, or between structures. Corticocortical and striato-striatal synchrony is stronger than cortico-striatal synchrony in WT and R6/2 mice (Fig. 2*C*, green lines). For Q175 mice, striato-striatal synchrony in the δ band is also strong (Fig. 2*C*, red solid line; only Q175 HOM are shown for clarity), and in the Q175 WT background is comparable to the R6/2 WT. For comparison, and as the null hypothesis, phase difference between random time series generated from Brownian motion do not show synchronization in the low frequencies (Fig. 2*C*, gray line). While δ band synchronization was observed, note that much of the δ band signal observed in striatum likely originated from cortex and was therefore recorded via volume conduction. This point is further developed in Discussion.

### γ Events occur at preferred phases of the δ cycles in HD animal models

**Figure 4.**
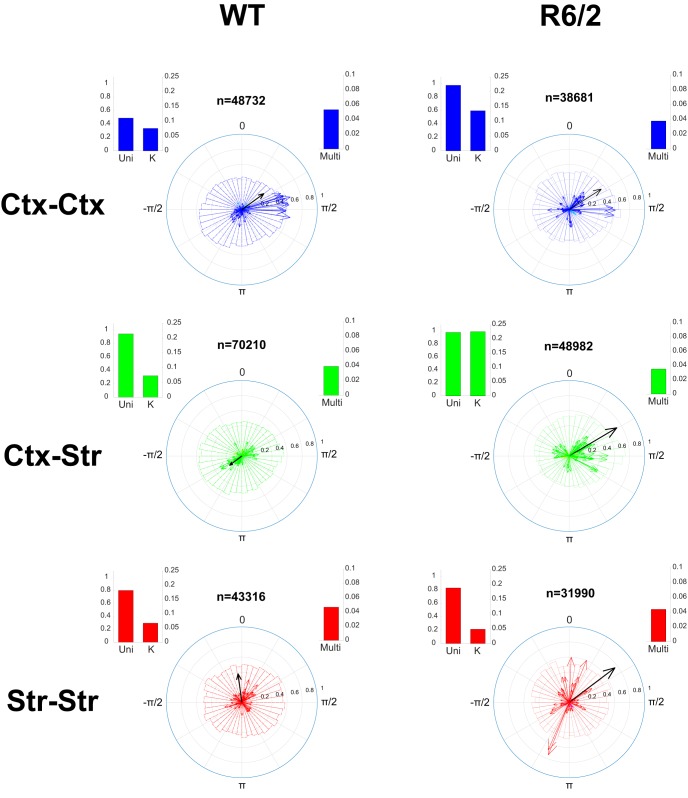
Phase-locking of gamma-events to delta rhythm in WT and R6/2 animals. Mean phase and strength of gamma-event phase-locking to delta oscillations is displayed in a polar plot for each pair of channels (one arrow/pair) across WT (left, 13 animals) and R6/2 (middle, 12 animals), drawn from an average total of 4 channels/animal. The mean vector across animals from the same strain is shown in black, and its norm is magnified by 10 to appear clearly with respect to individual pairs. Polar histograms show the distribution of all gamma events from all channels. Color-coded arrows represent the vector strength and orientation for each pair of channels, with statistically significant vs. insignificant (*p* < 0.05 vs. *p* > 0.05) phase-locking indicated by colors blue vs. cyan (Ctx-Ctx), green vs. yellow (Ctx-Str) and red vs. magenta (Str-Str). (Short arrows from insignificant vectors are nearly invisible). Inset histograms on top of each polar plot show (left) statistical tests for unimodality of the distribution (Uni) and the concentration of the distribution (K), and (right) the multimodality of the distribution (Multi) using the Hartigan’s Dip test (see methods).

**Figure 5.**
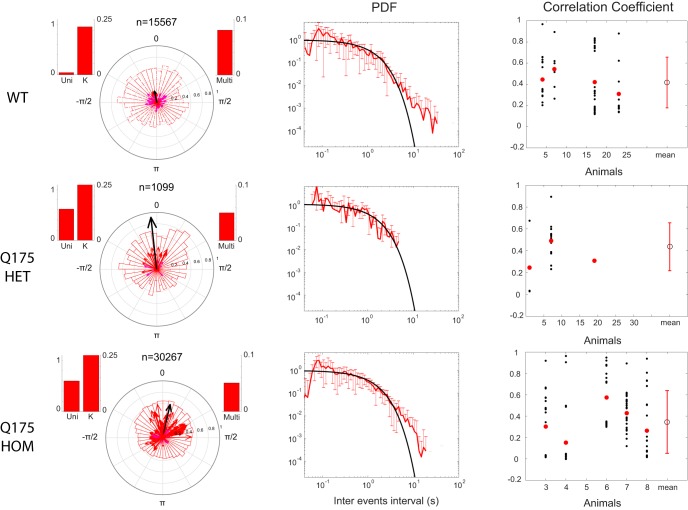
Phase-locking and inter-gamma events statistics in WT and Q175 animals. Same statistics as Fig. 3 and 4, computed for the Q175 animal strains. Left: Distribution of delta phases at each gamma event occurrence. Total number of gamma events extracted shown above polar plot as *n*. Histograms on sides of the polar plot indicate *p*-value of the statistical test for uni-modality (with concentration *K*) and multi-modality of the distribution, as computed by the Hartigan’s Dip Test (see methods). The mean vector across animals from the same strain is shown in black and its norm is magnified by 5 to appear clearly with respect to individual pairs. Middle: Mean probability distribution function (PDF) of inter-gamma events intervals across all electrodes from all animals. Standard deviation is indicated by error bars and a negative exponential is given as reference in black. Right: Correlation between gamma events for each electrode pairs (black circles) and mean (red circles) for each animal. Mean (open circle) and standard deviation (red bar) across animals from the same lineage are shown on the right-most of the x-axis.

The distribution of δ phases at which γ events occurred were compared for each channel pair and revealed stronger phase-locking of γ events to δ in HD animal models (Figs. 4, 5). Specifically, a unimodal circular normal distribution (i.e., a von Mises distribution) of δ phases at which γ events occur resulted in a large vector strength (Fig. 2*B*, left). The orientation of each vector represents the center of the γ-to-δ phase-locking and the length of each vector represents the probability that events occurred at or near the preferred δ phase. If all events from channels were perfectly phase-locked to the δ oscillation, the resultant phase vector average would have a norm of one and the length of the figure arrow would be maximized. Instead, a uniform distribution of phases results in scattered event-to-phase mappings and weak vector strength, with an arrow length close to zero (Fig. 2*B*, right). All circular distributions of γ events were assessed for non-uniformity using Rayleigh’s test and vectors corresponding to *p* > 0.05 are displayed more prominently (see Fig. 2*B* legend).

#### Cortico-cortical and cortico-striatal phase-locking distributions are narrower and unimodal in R6/2 animals while multimodal in WT

R6/2 phase-locking in cortico-cortical channel pairs (Fig. 4, blue) showed subtle differences compared to WT. The polar histograms revealed a multimodal distribution of phase-locking in WT with a peak at π/2 (corresponding to the downsweep of the δ oscillation) and another peak between -π/2 and π (corresponding to the early upsweep of the δ oscillation) which disappeared in R6/2. This difference was more prominent within cortex and striatum (Fig. 4, green). Channel-pair phase-locking was stronger (longer arrows) and more preferentially directed to the downsweep of the δ oscillation. Statistical tests for unimodal versus multimodal distribution confirmed this observation (bar histograms on upper corner of polar plots).

#### Striato-striatal phase-locking is stronger in HD animals than in WT background and its distributions more unimodal than multimodal

WT mice showed weak γ event phase-locking to δ phases in striatum, indicated by short, randomly directed vectors in Figure 4, red, bottom left. This likely reflects the absence of strong γ power in these animals and a possible noise source for detected γ events. Although stronger phase-locking is observed in some channel pairs recorded from R6/2 striatum (Fig. 4, red, bottom right), the absence of a clear peak in the distribution shown by the polar histogram indicates a lack of a preferred population direction across animals. However, the stronger γ-to-δ phase-locking is clearly present in the Q175 compared to WT mice (Fig. 5). Here, the population of phase-locked distributions was strongly unimodal and centered around 0 (±π/2 radians) corresponding to the positive peak of the δ cycle and constantly captured by the statistical test (bar histograms aside polar plots).

### Inter-γ events intervals indicate a random process

**Figure 6.**
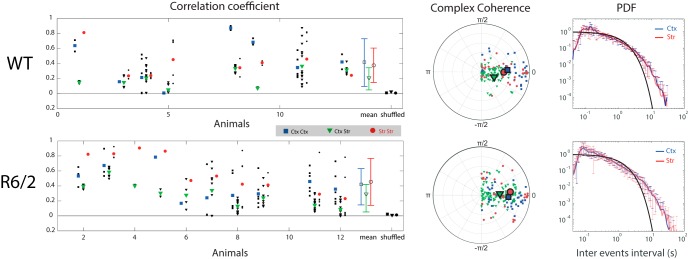
Gamma events have highly variable correlation across channels and brain regions. Left: Pearson correlation coefficients between gamma events from each pair of channels (black) within cortex (squares), between cortex and striatum (triangles), and within striatum (circles). The mean correlation within an animal across channel pairs is color-coded (blue for Ctx-Ctx, green for Ctx-Str, red for Str-Str). Population averages across animals and their standard deviation are denoted by open symbols (right, off-axis) using the same symbols and color code. Mean correlation coefficients of shuffled intervals are filled. Middle: Coherence of signals from each pair of channels during gamma events (same color code as above), averaged over the low gamma frequency band 25-55 Hz and projected in polar coordinates from the complex plane (see Methods). Scattered points showing spread in phase from 0 rule out volume conduction at gamma frequency. Right: Probability density function (pdf) of inter gamma event intervals in a log-log scale for each cortical (blue) and striatal (red) channels are plotted for WT (top), R6/2 (bottom). Black line represent exponential distribution for reference.

Having shown that γ events in striatum preferentially arise at specific phases of the cortical δ oscillation in HD model animals, and at a specific phase among the entire Q175 population, next we measured the regularity of striatal γ events (e.g., if they occur on every δ rhythm cycle or only randomly on some cycles). Note that this measure relates also to the uniformity of the distribution of levels of peak power in the fluctuating γ band-passed envelope. We assessed the regularity of γ events by inspecting the distribution of intervals between them, for each channel recording, across all animals. The inter-γ event interval distribution, for each animal model, and each recording region, falls between an exponential and a power-law distribution (Figs. 5, 6). An exponential distribution indicates a random Poisson process for event generation, whereas to generate a power-law distribution, the intensity of the Poisson process is not constant (see Chapter 8.1.7.2. in Croarkin et al., 2012) and results in a linear affine transformation on the log-log scale.

### Temporal correlation between γ events is higher within than between structures

The above analysis informs us that (1) δ oscillations occur on average at zero-lag phase differences across recorded brain regions; (2) striatal γ events in HD mouse models occur at a preferred δ phase, and across the Q175 population preferentially during the positive upsweep of the δ cycle; and (3) γ event genesis derives from a stochastic process. To test whether γ events depend on one another in their timing, we next computed the temporal correlations between γ events across channels from within and between cortex and striatum (Figs. 5, 6). Both high and low γ event correlation coefficients were observed between pairs of electrodes within and between cortex and striatum in all categories of animals, but on average, correlations within a structure were higher than between structures (Figs. 5, 6, mean, green triangles vs blue squares and red circles). These differences suggest that γ events in striatum are not strictly co-occurring with cortical γ events, although there exist previous examples of γ range oscillations being volume conducted several millimeters into the striatum from the piriform cortex (Berke, 2009; Carmichael et al., 2017), and θ oscillations from the hippocampus (Lalla et al., 2017). To further investigate this aspect, we computed the coherence between channels within cortex, within striatum, and across both regions (Fig. 6, middle panel). The imaginary part of the coherence shows non-zero values for a large number of channel pairs, indicating that low γ signal recorded from dorsal striatum and motor cortex may derive in part from endogenous mechanisms in each structure (although we observed that the imaginary coherence values approached zero when computed for strong γ events only). We also observed that the SD of the distribution of correlations is higher among HD than WT background mice. Differences between the distributions of correlation coefficients among electrode pairs in striatum (Figs. 5, 6, red circles) among WT, R6/2, and Q175 mice were further assessed by a two-sample K-S test (*p* = 0.05 between WT and R6/2; *p* = 0.00005 between R6/2 and Q175 HOM), which indicates these distributions were significantly different. These differences, together with the observed distribution of γ event intervals following a trend between the power-law and exponential distributions, suggested stochastic independent processes were contributing to γ events across recordings. We next aimed to account for these differences in a computational model, surmising that differences in cortical δ oscillations may differentially drive inputs to striatum required for γ genesis. In this case, the observed higher SD among correlation coefficients across channels can be interpreted as an indicator of more heterogeneous sources of excitation from cortical regions, modulated more independently in HD animals than in WT.

### Introducing electrotonic coupling via gap junctions in a striatal FSI network model is sufficient for γ genesis

**Figure 7.**
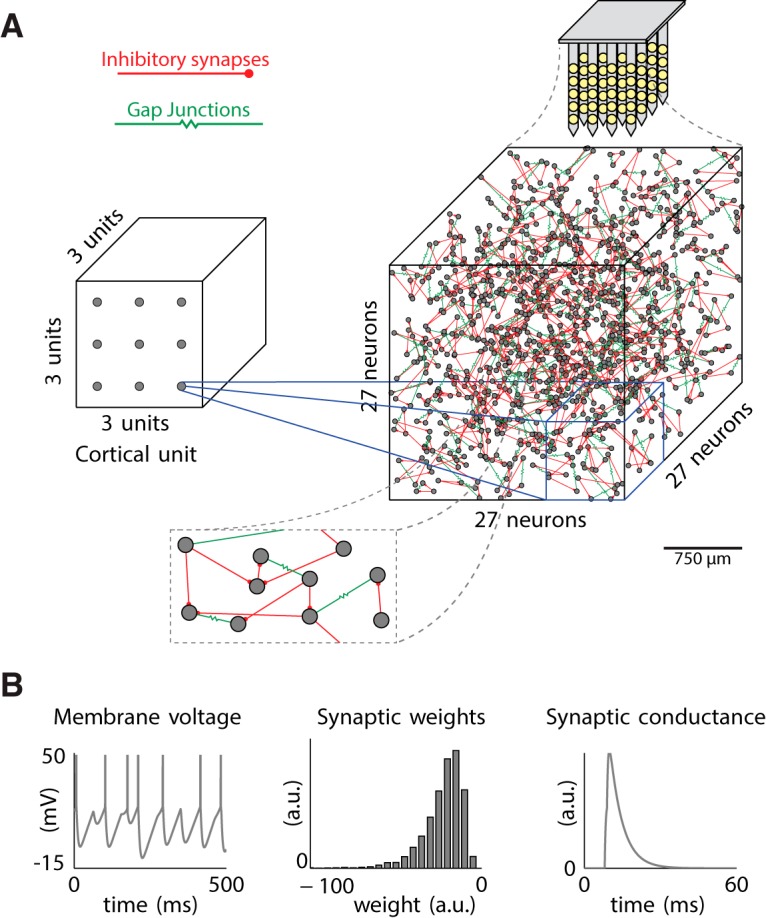
Computational model of FSI network and cortical drive. ***A***. Schematic of cortico-striatal FSI network model system. 27 cortical input are set in a 3 x 3 x 3 grid topographically projecting to a 27 x 27 x 27 grid of FSI units. FSI-to-FSI connectivity via gap junctions and GABAergic synapses is distance dependent (inset) and constrained by connection probabilities reported in the literature (Gittis et al. 2010). Extracellular fields are reconstructed by a modeled microelectrode array forming a grid of 64 channels (4x4x4) where each channel is spatially separated by ~750µm. ***B***. Example of single FSI unit membrane potential time series (left), distribution of inhibitory synaptic weights (center) and an exemplar time-series of a single IPSP (right).

We built a FSI spiking neuronal network (Fig. 7) with the assumption that the increase in γ power observed in the HD striatum is not exclusively derived from MSNs firing, although MSNs make up 90–95% of the striatal neuronal population. We reasoned that MSNs are less likely to participate in generating high frequency population oscillations because of their low intrinsic firing rate and slow membrane kinetics (Mahon et al., 2006; Miller et al., 2008). In contrast, FSIs are known to exhibit very high firing frequencies (Berke, 2011) and are connected to each other via gap junctions (Hjorth et al., 2009; Planert et al., 2010). Gap junctions are extremely fast conducting and have been shown to enhance synchrony both experimentally (LeBeau et al., 2003; Hestrin and Galarreta, 2005) and in studies of detailed network models of inhibitory neurons (Traub et al., 2001; Kopell and Ermentrout, 2004; Bartos et al., 2007). We implemented a network model of FSIs (see Materials and Methods) to assess which mechanisms might play a role in the observed γ band power increase and the γ event phase-locking to cortical δ rhythm.

**Figure 8.**
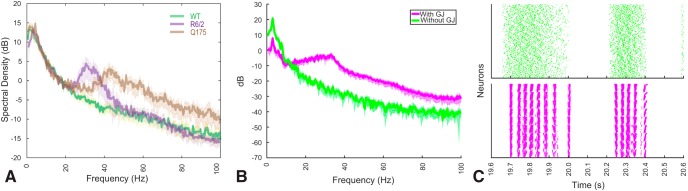
Spiking regimes of the modeled FSI network shows gamma-band population spikes embedded in the delta oscillation only when gap junctions are present. ***A***. Power spectra of striatal LFP from experimental data. ***B***. Power spectra of simulated LFP reconstructed from ~20,000 fast-spiking neurons (FSIs) with processed experimental cortical LFP, either without gap junctions (green) or with gap junctions (pink) between neurons. Shading denotes standard deviation. ***C***. Spiking raster plots of neurons in one *segment* of striatum (receiving drive from one cortical unit) either with (pink) or without (green) gap junctions.

We first configured our FSI network model with and without gap junctions (Fig. 8) and used the preprocessed experimental cortical LFP (see Materials and Methods) as input to the FSIs. Activity when gap junctions are absent is shown in a raster plot (Fig. 8*C*) and reveals an asynchronous mode of population spiking activity as also described elsewhere (Brunel, 2000; Traub et al., 2001). The mean individual neuron spiking frequency in this mode is controlled by the strength of the imposed cortical drive, which varies irregularly with the timing of positive fluctuations at the δ frequency. The power spectra reveal an absence of γ power in the population LFP, computed using a 3D Gaussian-dependent weighting of IPSPs as the recorded signal (see Materials and Methods). δ Power in the population LFP was wholly a result of cortical drive.

When spatially constrained gap junctional coupling is introduced into the model (Fig. 7; Materials and Methods), spike rasters show a synchronous mode of population spiking activity at the γ frequency. While the frequency of this synchronous activity can depend on network parameters, it is only weakly modulated by the strength of the cortical driving. γ Events are initiated by positive fluctuations at the δ frequency that exceed a threshold for γ genesis, and an increase in γ band power appears in the spectra due to synchronous spiking activity at this frequency, which slows at the end of each γ event, a phenomenon also observed in a model of transient θ modulation of γ band oscillations (Traub et al., 1996). Since cortical drive varies slowly at the δ frequency but with irregular amplitude fluctuations, this model generates robust γ events with irregular timing in the striatum. Despite its appealing simplicity, we demonstrate here that this model can account for multiple observations (found in HD) such as phase-locking of striatal γ events to driving δ band LFP.

### IPSP strength and total gap junction conductance determines γ frequency peak

**Figure 9.**
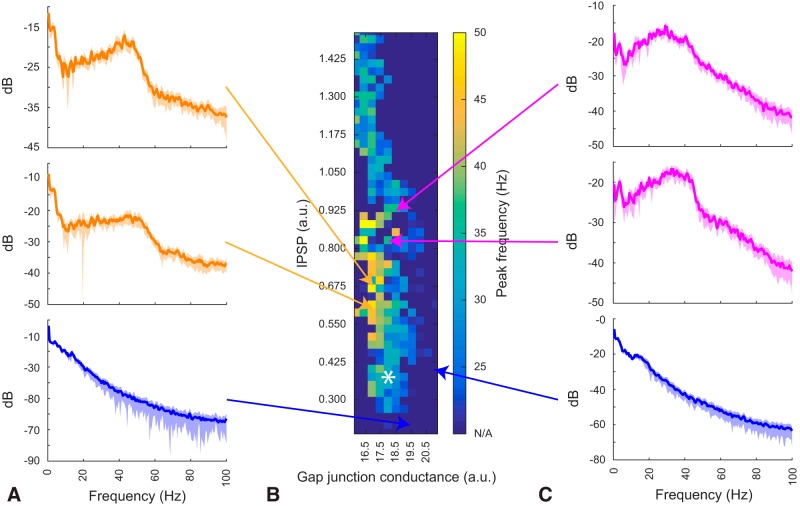
Gamma-band frequency peak shifts depending on the ratio between gap junction and synaptic coupling. ***A***. Example power spectra of simulated LFP reconstructed from ~20,000 fast-spiking neurons (FSIs), displayed for 3 different combinations of gap junctions versus synaptic coupling strengths. The peak in the 2 top yellow spectrum corresponds to power spectrum peak of Q175 animals from Fig. 1*B*, the blue spectra on bottom corresponds to a combination of parameters that does not produce a significant gamma oscillation. Shading denote standard deviation. The white asterisk (*) denotes the parameter combination used in the remaining of the paper. ***B***. Map of the parameter space exploration of gap junction coupling strength versus inhibitory synaptic strength in the FSI network model, where the color code quantifies the spectral peak frequency (>15 Hz). Blue entries corresponding to “N/A” represent simulations where either no gamma power was detected or the activity of the network was abnormal, e.g. completely silent or tonically firing at >100 Hz. All values are the mean of 10 simulations with random initial conditions. ***C***. Same as A) but the spectrum in pink corresponds to that observed in R6/2 animals (Fig. 1*B*).

We systematically varied FSI-to-FSI inhibitory synaptic weights (mean of the distribution) and gap junction conductance while maintaining the cortical drive from our experimental recordings as described above (Fig. 9). We found that different combinations of the two parameters resulted in the network oscillating at different frequencies (25–50 Hz). We explored this two-dimensional parameter space and found regions corresponding to R6/2 (γ peak at 31 ± 3 Hz) and Q175 (γ peak at 43 ± 3 Hz) animals. In the computational model, weaker gap junctional coupling combined with weak inhibitory synapses best reproduces the Q175 experimental data, while stronger gap junctional coupling or weaker gap junctional coupling combined with strong inhibitory synapses best reproduces R6/2 activity. Interestingly, a large increase in either gap junction conductance or inhibitory strength either reduces the peak frequency of γ power (Fig. 9*B*) or produces very fast tonic firing activity. We excluded these regimes for further analysis as the γ peak is too broad compared to the experimental data or the neuronal activity is not physiologic (i.e., tonic firing at rates >100 Hz).

### Cortico-striatal coupling strength alone can explain low or high γ power when synaptic couplings are fixed

**Figure 10.**
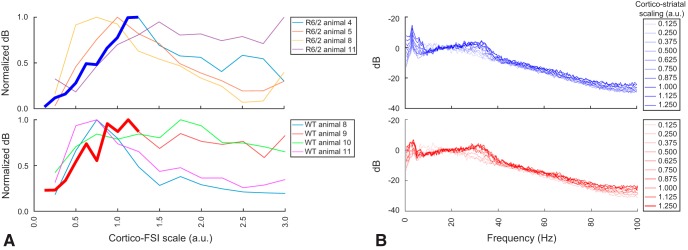
Cortico-FSI coupling strength can transition network from WT to HD gamma phenotype. ***A***. Gamma power (averaged over the 25-55 Hz band of the spectra) of simulated LFP for different cortico-FSI coupling gain levels, with input drive taken from 4 different R6/2 (top) and WT (bottom) animal recordings. Bold traces correspond to spectrum shown in ***B***. ***B***. Power spectrum from simulated FSIs LFP with increasing strengths of cortical drive extracted from R6/2 animal 4 (top) and WT animal 9 (bottom) recordings.

**Figure 11.**
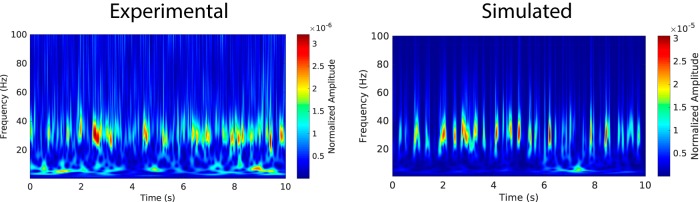
Time-frequency comparison between model and experimental LFP. Wavelet analysis of the LFP from experimental recordings (left) and reconstructed from the model (right), computed between 0 and 100 Hz (step size: 0.5 Hz) for a random epoch of 10s of quiet rest. Wavelet coefficients are normalized to sum at 1. LFP experimental time series taken from R6/2 animal 10 striatal recording, and simulated LFP with scaling factor CtxFSI=1.

We next used the model to explore whether impairment or enhancement of cortico-striatal coupling is sufficient to explain the increase in HD γ power. We systematically varied cortico-FSI coupling (by decreasing or increasing the gain of the cortical drive) while maintaining the cortical input statistics, IPSP weights, and gap junctional conductance constant in the model, at the value indicated by a white star in Figure 9*B*. As cortico-FSI drive is increased, our network transitions from weak to stronger γ power (Fig. 10), reproducing the observation from the experimental spectra (Figs. 1, 8*A* ). The increase in γ power is correlated with the strength of the cortico-FSI drive for weak to medium cortico-FSI coupling strengths but further increases to the cortico-FSI drive reduce the γ power. The time-frequency analysis applied to an experimental trace and the model indicate faithful replication of the observed phenomena (Fig. 11). γ Power changes were consistent in the model regardless of whether the processed experimental cortical LFP from WT or HD (R6/2) animals was used as the network stimulus, or whether we measured the change at the R6/2 frequency peak (31 Hz) or Q175 frequency peak (43 Hz). This result argues against abnormal cortical activity alone being necessary for the HD phenotype to emerge since the model exhibits WT or HD dynamics with either the WT or HD filtered cortical LFP as input.

### γ Event statistics in HD, but not absence of phase-locking in WT, explained by the simple FSI network model

**Figure 12.**
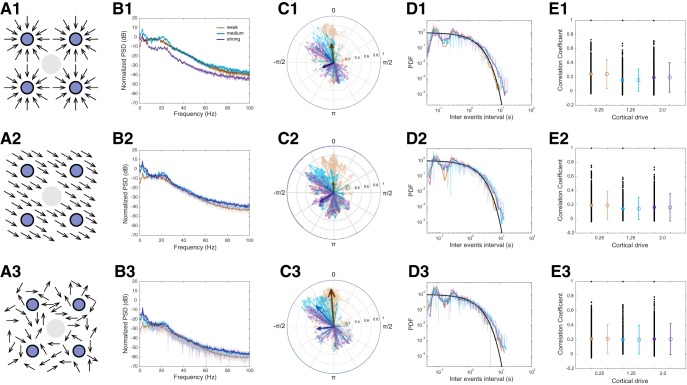
Statistics of the model for different cortical drives and LFP composites. ***A***. Illustrations of different LFP composites (blue circles: electrodes; gray circle: ground). A1) All units’ fields near electrode point towards it; A2) All units’ field point in the same direction; A3) Each units’ field orientation is random. ***B***. LFP power spectra for different cortical drives (weak: CtxFSI=0.25; medium: CtxFSI=1.25; strong: CtxFSI=2.0) for each LFP composite presented in A1-3. ***C***. Delta-to-gamma phase-amplitude coupling of the computational model with weak (brown), medium (blue), and strong (purple) cortico-FSI drive (*see Methods*). 0 portrays the top and –π/2 the upsweep of the delta oscillation. The mean vector across electrodes from the same cortico-FSI drive is shown in bold and its norm is magnified by 2 to appear clearly with respect to individual pairs. Shown for each LFP composites illustrated in ***A***. ***D***. Probability density function estimate of the inter gamma events interval distribution of LFPs from the model for increasing cortico-FSI drive (same color code as in ***B*** and ***C***), for each LFP composite from ***A***. Black line indicate exponential distribution for reference. ***E***. Pearson correlation coefficient between gamma events from different reconstructed electrode signals (one black dot per electrode pair), for the three combinations of cortico-FSI drive (weak: brown; medium: blue; strong: purple) and LFP composites (E1-3). Mean and standard deviation for each driving strength are indicated by circle and error bars on the right.

To compare simulated results from the computational model to the experimental data, we extracted the same statistics reported for the experimental data, i.e., γ event phase-locking to δ, correlation between γ events across reconstructed LFPs, and the inter-γ event interval distributions (Fig. 12). LFPs were reconstructed using a 3D Gaussian kernel around several points in space representing the recording electrodes, where the contribution of each unit to the field is calculated based on its distance to the electrode and the orientation of the electrical field it generates (computed as the sum of synaptic and active membrane currents, see Materials and Methods). Since the neuronal contribution to the LFP is a strong subject of debate in an open-field system such as the striatum (see Discussion), we examined the LFP spectra generated by the network composite according to 3 scenarios (Fig. 12*A*): (1) all units’ field point toward electrode; (2) all units’ field point in a unique direction; and (3) each units’ field point to a random direction. Regardless of which of this composite is used to compute the LFP, we observed that our computational model reproduces the γ event correlations observed in HD animal models, and fluctuations in δ band cortical LFPs were sufficient to drive the model to reproduce inte-revent interval distributions (Figs. 4–6, 12*D*). However, phase-locking properties could only be replicated for the HD phenotypes, where inter-electrode phase-locking of γ events to specific δ phases occurs (Fig. 12*C*). Inputs strong enough to generate γ in our model also always produced δ phase-locked γ events, and as cortico-FSI drive was further increased, the model phase-locking shifted from a peak at ∼0 to a peak at -π/2 radians. The computational model with weak cortio-FSI drive, therefore, corresponds best to Q175 striatal recordings in which γ events are phase-locked to the top of the δ wave (Fig. 5, bottom). Our model is also compatible with some strongly phase-locked distributions from the R6/2 phenotype (Fig. 4, right column), although the preferred δ phase distribution differs (experimental R6/2 phase-locking distribution peaks at π/2, i.e., in the downsweep of the δ wave, while our model peak at -π/2, the upsweep of the δ oscillation). Also, the more uniform distribution at which γ events occur with regards to the δ oscillation observed in WT that could not be reproduced by our FSI network alone (Fig. 12*C*). Such discrepancy suggests that although FSIs may explain the increase in γ power observed in HD and some aspects of the phase-locking, modifications or extensions to the model may be required to reproduce the full range of electrophysiological phenotypes in HD and WT. For example, other cell types, physiologic coupling, noisier input, more heterogeneous neuronal properties, or cell autonomous mechanisms not captured by our model may contribute to the dispersion of γ events across δ phases.

## Discussion

Our analysis revealed that δ band synchronization is unaffected in HD, but that γ events in striatum cluster at the peak of the δ wave in contrast to their more uniform or multimodal distribution over the entire δ wave in WT. We also demonstrate, using our computational model, that gap junctions strongly facilitate the generation of γ oscillations in the striatal FSI network.

### Sources of δ and γ band oscillations

We suggest that synchronized cortical sources may be stimulating the striatum at the δ frequency, and that by this driving mechanism, the striatum may abnormally “ring” at the γ frequency in HD on particularly strong cortico-striatal volleys. This view is supported by cortical evidence that in a slice preparation with NMDA, AMPA/kainate, and GABA-B receptors blocked, a transient puff of a mGluR agonist evokes interneuron network γ activity (Whittington et al., 1995). In addition, intrinsically bursting L5 cortical pyramidal neurons are likely responsible for generating cortical δ band oscillations (Carracedo et al., 2013; Shepherd, 2013). In subjecting our model to the same PAC analysis as our experimental data, we find that by decreasing the gain of δ cortical input to the FSI network, absolute γ event genesis subsides, albeit still strongly phase-locked to the peak of the δ phase. Thus, motor cortical inputs to a model FSI network alone cannot reproduce the more heterogeneous distribution of γ locking to δ phases observed in WT mice.

### PAC methodology

We analyzed γ-to-δ phase synchrony and phase-amplitude relationships in simultaneous brain recordings of HD mouse models versus WT. Our dataset comprised multiple channel recordings from the striatum (WT, R6/2, Q175) and motor cortex (WT and R6/2), with a focus on periods of quiet rest. We developed a method to extract transient increases in γ power (i.e., γ events; Fig. 2) from a channel, and computed their relation to the instantaneous phase of δ band oscillations from another electrode. Our technique resembles the one described by Voytek and colleagues (Voytek et al., 2010, 2013) but differs slightly from others (von Nicolai et al., 2014; Seymour et al., 2017) in that we use a single Hilbert-transformed signal for the phase of slow oscillations rather than multiple band-passed filtered signals (of width ∼1Hz) commonly used when the phase signal is in higher frequency bands such as α. Also, rather than assessing the whole range of γ amplitudes with respect to δ phase, our method focuses on γ power transients by extracting statistically strongest γ power. Extracting such transient γ events from signals’ statistics is advantageous when the γ power is strong over the whole recording (in contrast with task-related PAC) and thus present at all phases of the slow frequency (otherwise the γ transients would be “blurred” by averaging with strong background γ power). On the other hand, our technique suffers the pitfall of detecting γ events even when γ power is weak, and thus less meaningful fluctuations in γ amplitude may be interpreted as γ events. Nonetheless, in the case of γ event signal being pure noise, the distribution of their δ phases would be uniform (i.e., von Mises, as observed more in WT). Despite weak γ power in heterozygous Q175 (Fig. 5), the technique detects strong phase-locking that is statistically significant (*p* < 0.05). We showed that γ events in all HD animal models are more strongly phase-locked to some phases of the δ oscillations recorded from dorsal striatum and motor cortex (Figs. 4, 5), yet do not occur at every cycle (Fig. 6).

### Phase difference in δ oscillation

We suggest that δ oscillations observed in striatum are transmitted as feedforward excitation from cortex because, on average, δ oscillations display zero-lag phase synchrony across all simultaneously recorded channels in cortex and striatum (Fig. 2*C*). However, δ band phase differences between motor cortex and striatum in WT and R6/2 are more widely distributed than within striatum, suggesting more variability in cortico-striatal δ phase differences than within-cortical and within-striatal δ phase differences alone. This observation is consistent with a previous study using the same dataset for WT and R6/2 mice (Hong et al., 2012) in which near-zero lag synchrony was observed in R6/2 but not in WT (where phase differences peak at 20 degrees). In this previous analysis, the relative phase differences were computed based on mean coherence, which is performed in the frequency domain and assumes that the phase difference is stationary over time. In the present analysis, we compute the phase differences in the time domain using the instantaneous phase of the signal given by the angle of the Hilbert transform of the signal when projected into the complex place. This measure better captures the non-stationarity of the phase relationship and is closely related to the phase-locking value (PLV) first described in Lachaux et al. (1999), where instantaneous phase synchrony is measured as the absolute value of the exponential of the phase difference. The PLV is often averaged across many trials in cognitive tasks involving the analysis of evoked-responses to assess the increase/decrease of synchrony between brain regions as a measure of functional connectivity (Aydore et al., 2013). As our analysis involved only spontaneous activity during rest, we simply used the phase difference.

### Possibility of γ originating from cortex

Our correlation and complex coherence analysis of experimental recordings also suggests that γ oscillations observed in striatum are unlikely transmitted from cortex (Fig. 6, green triangles), although some recorded signals from striatum can overlap spatially with a proximal cortical source due to volume conduction (Berke, 2009; Carmichael et al., 2017; Lalla et al., 2017). It is a generally agreed principle that γ is related to local processing versus lower frequency bands which are more akin to long distance signaling (Kopell et al., 2000; von Stein and Sarnthein, 2000). However, γ synchronization may also exist over long distances in some attentional tasks in humans (Gregoriou et al., 2009) and although our analysis comprises only electrodes in motor cortex (for WT and R6/2), cortico-striatal coupling in the γ band may exist, especially in rodents where cortico-striatal projections are at most 1- to 1.5-mm long and axonal velocities range from 0.5–3.5 m/s in mice (Swadlow and Waxman, 2012), Theoretically, this should give rise to only 0.3- to 2-ms conduction delays in cortico-striatal signaling (well within the γ period). The high variability of correlation coefficients and imaginary coherence between channel γ events suggests either a spatial segregation of variable patches of neural activity in the γ band or distant synchronization between patches (Lindén et al., 2011). For example, one could predict either a high correlation among recordings from close electrode proximity in which two distinct channels record the same population of neurons, or high correlation among distant electrodes in which distinct striatal (or cortical) regions have very similar activity. For the latter, two areas can either receive the same inputs or share internal constraints limiting them to the same dynamical regime (assuming similar neuronal substrates for information processing throughout the structure). In this case, the latter possibility can be further investigated using the computational model and exploring the role of synaptic strengths and its effect on the size and length of spatiotemporal structures formed by the network of FSIs and recorded LFPs. Low correlations and coherence, whether between proximal or distal electrodes, indicate either different network processes or dynamics operating on the same input, or two striatal (or cortical) regions processing different inputs. Although the latter seems more realistic in view of the homogeneity of the neuron types and network structures within the striatal (or cortical) microcircuitry, the former should not be ruled out. In the context of cortico-striatal projections, which is our focus here, regardless of electrode positioning, the interpretation remains unchanged: on average, γ event correlation is stronger within a structure than across structures.

### Interpretation and modeling of LFP signal

The spatial reach of LFPs is still a subject of debate, and several recent forward modeling studies have tried to address the biophysical substrate of those recorded signals (Lindén et al., 2011; Einevoll et al., 2013; Teleńczuk et al., 2017) albeit mainly focusing on cortical LFPs. Indeed, the current-source density paradigm to reconstruct current sinks and sources works best when recorded regions contain neurons with elongated dendritic trees, such as L5 pyramidal cells, receiving excitatory inputs on apical dendrites and a mixture of inhibitory and excitatory inputs on basal dendrites. Such configuration forms an open-field structure giving rise to strong dipolar and possibly quadrupolar LFPs (Pettersen and Einevoll, 2008), which amplitude decays by 1/r^2^: the square of the distance to the current source (Destexhe and Bedard, 2013; Reimann et al., 2013). In contrast, closed-field structures such as networks of stellate cells do not have preferred orientation or spatial pattern of synaptic inputs, giving rise to smaller monopolar LFP contribution because transmembrane currents tend to cancel each other out, and the field amplitude then decay in 1/r. In this respect, the striatum would fall in the second category as its neuronal populations (MSNs, FSIs, low-threshold spiking or cholinergic interneurons) each display rather random spatial orientations of dendrites. This raises questions about the origin of signal recorded from striatal electrodes implanted close to cortex as electric fields most certainly propagate from neighboring cortical regions or the hippocampus (Lalla et al., 2017), and we think it might be the case especially for high power low frequency bands such as δ and θ given the low-pass filtering properties of extracellular medium (Bédard et al., 2004, 2006; Destexhe and Bedard, 2013).

The depth of electrode implants may also affect the interpretation of the recording, as it is known that upper cortical layers reflect afferent inputs (i.e., thalamic drive) while electrodes in deeper cortical layers are more disposed to record efferent cortical signals (Buzsáki and Schomburg, 2015). Artefactual phase-locking in the high-γ (>80 Hz) band has previously been reported due to muscle activation using electromyography (Buzsáki et al., 2012), but we have not considered this contingency since our analysis is restricted to low-γ frequency bands (25–50 Hz).

### Relation to pathophysiology and behavioral states

In case of a purely volume-conducted signal from nearby cortical regions (such as the sensorimotor cortex), the interpretation of our analysis would apply to a cortical-only source of γ, most likely generated by the interaction of pyramidal cells (PY) and interneurons (IN), specifically via perisomatic inhibition of PY by parvalbumin-positive (PV) IN (Buzsáki and Wang, 2012). Intriguingly, the consequences of such an interpretation on behavioral aspects would be highly relevant to HD pathophysiology: sensorimotor γ oscillations have been related to active walking in humans (Seeber et al., 2015), with distinctive functional processes related to movement initiation and sustained movement in high (75–100 Hz) and low (35–50 Hz) γ bands, respectively, in a visuo-motor task (Crone et al., 1998). Our data analysis also supports a disruption in cortically generated γ oscillations (Fig. 1, blue traces; and Fig. 4, top row), but our computational model architecture does not explicitly account for excitatory spiking populations interacting with the FSIs. Hippocampal γ, on the other hand, is less likely to occur at rest in healthy condition, and its cross-frequency coupling is usually associated with memory recall in goal directed tasks in rodents (Johnson and Redish, 2007; Tort et al., 2008; van der Meer et al., 2010), which is not congruent with HD symptomatology.

Finally, our analysis focused on the electrophysiological properties of the cortico-striatal system when the animals were at quiet rest because increased γ power is observed in HD animals as compared to WT in this behavioral state. Previous studies have also reported altered power spectra in the β band during grooming (Hong et al., 2012) and sleep (Jeantet et al., 2013) among HD mouse models, but we speculate that such β disruptions have more to do with the pallido-thalamo-cortical system than the cortico-striatal system alone. Indeed, β oscillations have been implicated in the performance of motor behavior and evidence has suggested β genesis as strongly related to thalamo-cortical projections (Sherman et al., 2016) or the pallido-subthalamic loop (Lienard et al., 2017), supporting our view that altered β rhythm is best associated with basal ganglia microcircuitry and its afferent rather than efferent projections.

### Scope and predictions of the computational model

Our computational modeling of an FSI network revealed that altered cortico-striatal drive to this network is sufficient to explain the power increase in the low γ band with respect to other frequency bands as observed in HD mice. The model further predicted that the extent of γ power is correlated with the strength of cortico-striatal drive, a prediction that is testable experimentally. We processed experimental LFPs from both WT and HD animals while keeping intrastriatal couplings constant without detecting significant differences. This suggests that neither stipulating changes in cortical activity nor stipulating intrastriatal circuit changes are required to increase striatal γ power with respect to other frequencies.

We developed our FSI computational model based on previous seminal modeling work from four neuronal populations within cortex (Traub et al., 2001), and the supporting observation that FSIs are by far the most prominent neuronal subtype for generating γ band oscillations. One could argue that our FSI model lacks realism due to its simplified membrane dynamics and lack of an exhaustive set of ion channels known to play a role in dendritic processing and neuronal excitability. Our choice of an IAF model, however, derives from the scope of our study. We aimed to investigate network properties of inhibitory FSIs and not single-neuron properties, which rely on dendritic morphologies, ion channel kinetics, and other intracellular mechanisms. We selected a parameter range for our modified IAF model that displays type 2 excitability, i.e., exhibiting a jump from zero to a finite firing frequency (Ermentrout and Terman, 2010; Gerstner et al., 2014). Additionally, the frequency-current curve (F-I curve) of our model matches one generated by a much more detailed FSI model incorporating neuron morphology and 127 compartments into a conductance-based model (Hjorth et al., 2009), further supporting our use of the greatly simplified model for point neuron network computations.

Fast and strong inhibition in inhibitory neuronal networks, mediated by GABA_A_ synapses via shunting, is a promising candidate for γ genesis (Bartos et al., 2007). The presence of gap junctions, which are estimated to affect the cell membrane potential by 2–11% (Hestrin and Galarreta, 2005), is known to have homogenization properties at the network level and enhance coherent firing (Bartos et al., 2007). Field effects also exist in so-called ephaptic coupling (Anastassiou et al., 2011), which is instantaneous, although more isotropic when propagating. An increase in ephaptic coupling would be consistent with observations of high neural loss in striatum, mediating stronger field potentials as opposed to a densely packed neuronal substrate with higher impedance.

### Necessity for integrated models of striato-nigro-pallidal microcircuits

We showed that an increase or decrease in cortico-striatal drive can account for an increase in low γ band power with respect to other frequency bands and that the statistics of our model agrees with the experimental data, but the model could not account for the uniformity or multimodality of γ event-δ phase distributions observed in WT mice during rest. This suggests that other mechanisms are at play under normal conditions to generate a uniform/multimodal γ event to δ phase. Further modeling, including additional sources of noise and heterogeneity or coupling the FSI network model to a model of the MSNs network (Ponzi and Wickens, 2010, 2012, 2013) might address these points. In fact, a recent study (Wu et al., 2017) demonstrated the feasibility of low-γ band oscillations emerging from a network of MSNs alone, and the presence of FSIs coupled by synaptic and electric coupling amplified those oscillations. Another rigorous study of FSI-MSNs interactions will add to the picture in a subsequent publication (Chartove et al., 2017), but note that to date, even the larger computational models of the basal ganglia circuitry cannot scale to realistic numbers of neurons and synapses (Lindahl and Hellgren Kotaleski, 2017) to compute the LFP. Also, there are important structural and functional distinctions between striatal subfields (Berke et al., 2004; Berke, 2009) that are not accounted for in those current modeling studies. It will be important to address these differences in future studies.

Finally, McCarthy et al. (2011) showed that β range frequencies up to 20 Hz can be reproduced in a MSNs network endogenously when non-inactivating potassium currents (M-currents) are present, shedding light on the likely importance of rebound spiking mechanisms for the genesis of abnormal oscillations in purely inhibitory systems with disrupted dopamine, a conditioned encountered in Parkinson’s disease. To this respect, we could not address here any of the question that remains in HD regarding dopamine abnormalities (Rangel-Barajas and Rebec, 2018). The classic view that HD pathology is due to loss of D2- MSNs has been revised in the past years, as it was demonstrated that D1-MSNs are also affected early in the disease (André et al., 2011; Galvan et al., 2012; Chen et al., 2013). The interplay between D1 and D2 MSNs in disease evolution is unclear (Raymond et al., 2011; Bunner and Rebec, 2016), and its time-dependence with Glutamate disruption is rather tenuous (André et al., 2010). What is clear is that dopamine is affected in HD, but our analysis and modeling cannot address dopaminergic components as the focus is on cortico-striatal fast-slow oscillations and γ genesis among striatal FSIs rather than striato-nigral modulation.

## Conclusion

Our analysis revealed that cortical δ band activity alone is not different between WT and HD animals, but that the PAC of striatal γ events to the δ oscillation are modified in HD. Assuming that the main input to the dorsal striatum originates from cortex, we used experimentally recorded cortical signals to drive a computational model of striatal FSI network, and inferred that the strength of cortico-FSI synapses proportionally account for the γ power observed in striatum, while the balance of gap junction conductance and IPSP strength determine the γ peak frequency.

We suggest that striatal γ is a marker of abnormal cortico-striatal processing which can be interpreted in two ways. (1) γ Genesis is pathologic by itself and causes the disturbance: the fast striatal γ oscillations act to bias striatal spatiotemporal processing toward pre-defined activation patterns which are cortico-dependent. Disturbances in these activation patterns results in discordant downstream basal-ganglia processes propagating to thalamo-cortical systems influencing cognitive and motor operations. (2) γ Genesis is a compensation mechanism that permits striatal processes to be maintained albeit the disruptions in cortico-striatal processing: the activity bias imposed by fast γ oscillations in response to stronger cortical drive emerge to try to keep control of downstream basal-ganglia circuits, those downstream circuits being altered in HD and causing cognitive and behavioral operations.

At the current stage, neither the recorded data nor the FSI computational models are able to disambiguate between those two scenarios. A larger circuit model integrating FSIs and MSNs with direct and indirect pallidal pathways would be required to investigate those two hypotheses, and new experimental recordings covering other entities of the basal ganglia nucleus would help in the disambiguation. Specifically, a recent study using calcium imaging has confirmed the existence of spatiotemporal activity patterns in striatum encoding action space (Klaus et al., 2017), and such technique applied to HD mice models could shed light on the functional nature of the activations patterns in striatum related to the pathology. One experiment to test our predictions might involve selectively blocking MSNs spiking in awake resting WT and HD mice at symptomatic age. A recent study by Klaus and Plenz (2016) selectively removed all GABAergic transmission in striatum, or blocked synaptic excitation onto FSIs only, then measured consequences of the pharmacological blockade among cortical spiking statistics (i.e., avalanches), striatal spiking correlation, and movement initiation. In our proposed experiment blocking spiking among MSNs only and not FSIs would match closely the microcircuit model used in our simulations. Furthermore, in awake animals adding multiunit recordings would allow interpretation of spiking statistics with respect to cortical slow oscillations, and further disambiguate volume-conduction versus local microcircuit sources.

10.1523/ENEURO.0210-18.2018.sm1 Supplementary 1 Supplementary Analysis & Modeling code. Download Supplementary 1, ZIP file

## Synthesis

Reviewing Editor: William Stacey, University of Michigan Decisions are customarily a result of the Reviewing Editor and the peer reviewers coming together and discussing their recommendations until a consensus is reached. When revisions are invited, a fact-based synthesis statement explaining their decision and outlining what is needed to prepare a revision will be listed below. The following reviewer(s) agreed to reveal their identity: Daniel Leventhal, Matthijs van der Meer.

The reviewers both were quite interested in the results, but brought up several questions and concerns. I refer to the comments below for specifics, which I have edited to reflect the synthesis conversation we had.

There are a couple of specific items that should be prioritized, but I suggest you go through each of the comments below as well.

1. The connection to, and effect of, Huntington's disease needs to be strengthened.

2. The model of LFP that you chose may not have been appropriate, and since that research area is such a huge unknown and the results are so heavily influenced by this model, either that part should be redone or it should be better justified.

3. That issue of LFP modeling is more important because it is very important to make a distinction between rhythmic activity in local spiking and synaptic currents versus the LFP, which is a big question for this discipline. The conclusions about mechanisms of the waveforms may be dependent upon your model, and since that model might not be valid for these neurons it calls the conclusion into question.

Reviewer 1:

The authors present an interesting study that elaborates on previous findings regarding LFP dynamics in corticostriatal circuits in animal models of HD. The model nicely complements the experimental results (mainly that striatal gamma events are more tightly locked to delta oscillations in HD mice) to suggest new hypotheses to be tested in future experiments/models. I have several methodological questions, mostly centered around the data analysis, described below. My major high-level comment is that a lot of time is spent characterizing differences between WT and HD mice, and then suggesting (or refuting) potential neuronal mechanisms to explain them using the model. What is missing, in my opinion, is a link between these findings and the pathophysiology of HD (or why use HD mice at all?) Both the introduction and discussion could be enhanced by discussion of how these electrophysiological differences may be related to the underlying pathology of HD and/or the clinical phenomenology of HD.

More specific comments:

Any LFP study could be confounded by the choice of reference. Are the 50 micron ground electrodes also the reference signals? If so, are all cortical channels referenced to the cortical ground and all striatal channels referenced to the striatal ground?

One of their major conclusions was that the physiologic abnormalities could result from reduced cortical drive to FSIs. The “classic” HD pathology is loss of D2 MSNs (I'm not sure what the pathology looks like in these models), though of course there is widespread pathology especially with progression. That may be a point worth making - that their model suggests that this specific abnormality should be correlated with the degree of cortical pathology?

I also agree with Reviewer 2's point about using gamma events to demonstrate coupling. I'm used to various other measures of phase-amplitude coupling (de Hemptinne et al, 2015, Canolty et al, 2006, among others) that look at high frequency power as a function of low frequency phase. I think their conclusions are probably accurate, but it's not clear to me that they detected isolated gamma events, or could they have detected broadband power increases? Figure 2 looks like they're detecting true gamma events, but that was part of my logic in asking for representative samples from each strain - are these similar events? I think this is similar to R2's point about matching behavior between strains.

Lines 142-144 and 151-160: two definitions of phase are given: theta(t) = Im(H(x(t))) or arctangent of the ratio of the imaginary to real part (which is the definition with which I'm familiar). Is there a utility that I'm missing in having two definitions?

Lines 148-159: “The measure of amplitude modulation is given by the average correlation coefficient across the analytic amplitude of each channel, resulting in a value in the interval [-1 1].”

I'm not clear what values are being correlated. Is this a correlation between Aj(t) recorded on different electrodes? If so, it is not clear to me why that would be a measure of “amplitude modulation.” Is this amplitude modulation across space? Related to this question, what is the inter-electrode separation within each bundle?

Line 162: higher standard deviation of what quantity? The difference between actual frequency and frequency calculated by the linear fit (pj(t) - pj(t-1))?

Line 166: Why did this calculation use theta ( Im(H(x(t))) ) instead of Pj(t)? Perhaps I'm missing something obvious, but again it's not clear to me why there are multiple definitions of phase.

Lines 369-371: Given the zero phase lag between cortical and striatal delta, is it possible this is a volume conducted signal from one region or the other (or another region entirely)? I don't think this would fundamentally change the results, but should probably be noted somewhere.

Is there a qualitative difference between gamma events in each mouse strain? For example, in Figure 2A, which mouse strain did the examples come from? Do identified events look different in different strains? As the authors note (lines 190-192), for signals with weak gamma power, extracted events could represent “noise.” If this is the case, couldn't the gamma-delta phase locking results just be a consequence of the fact that there isn't enough gamma power in WT mice to detect true gamma events? This possibility has obvious implications for interpreting the result that the relatively uniform distribution of gamma-delta phase locking in WT mice is not reproduced by the model (lines 529-536).

In Figure 3 (and Figure 5), why are some of the animals missing? Too few channels to compute correlations?

In Figure 4, the color coding of the mean vectors is difficult to see. Could the authors use colors with greater contrast or some other method to make these plots clearer?

In the section on temporal correlations within/across structures (starts on line 424), there is a lot of discussion of the spatial distribution of gamma events. Is it possible to correlate inter-electrode distance with the temporal correlation of gamma events? Or not enough variance in electrode separation to do that?

Can the authors estimate the layer in which cortical electrodes reside? There should be a phase reversal in LFP phase as electrodes traverse cortically generated dipoles.

I think in general it is problematic to use LFP phase differences to determine whether one region leads/lags/is synchronous with another (argument starting on line 559). The phase of cortical oscillations depends on electrode depth. So I would expect the phase difference between cortical and striatal LFPs to change depending on the depth of the cortical electrodes. Also, if the LFPs are measured against different references, that could change the phase relationship.

Minor point:

Line 181: {x} should be &lt;x&gt;

Reviewer 2:

This study seeks to advance our understanding of corticostriatal circuit dysfunction in Huntington's (Htt) mice. By analyzing a substantial body of previously published data, the authors find altered patterns of local field potential oscillations in the dorsal striatum, and altered coordination with cortical field potentials. Using a point neuron spiking model whose activity is converted to simulated LFPs, it is suggested that these abnormalities are compatible with reduced cortical drive of striatal FSIs.

Overall, the topic addressed here is clearly important, not only because of the long-term potential for clinical advances, but also because Htt serves as a useful model for how translational neuroscience can confront the challenge of dissecting the systems-level consequences of a single genetic mutation. Much work in Htt has focused on a single level of analysis only (e.g. synaptic physiology or behavior); in contrast, a real strength of this paper is that the authors bring together experimental data, sophisticated data analysis, and modeling in a single study.

The main weakness I see is that currently, in the field as a whole, the connection between the striatal LFP and local striatal circuits and circuit elements is rather tenuous. As a result, I think it is not as straightforward as the authors might like to relate the LFP data they analyze to inferences about underlying circuits. This overall issue is by no means unique to this study, and most of the work the authors report here can still be useful. However, it does limit the strength of the conclusions that can be drawn, and I think the authors need to do a better job of being completely transparent about this. At a minimum, clarifications should be made to the text, but even better would be to improve the model component that simulates the LFP given spiking activity with a version that captures striatal neuron morphology more accurately. That is because the model of LFP generation may not be correct for these striatal cells: MSNs and FSIs are much more like stellate cells than they are like pyramidal cells (see below)

Major comments:

Overall, I think the manuscript would benefit from a more explicit distinction between rhythmic activity in local spiking and synaptic currents on the one hand and in the LFP on the other. As the authors appropriately discuss, it is currently unclear to what extent LFPs recorded from the striatum reflect transmembrane currents that originate in the striatum. Yet, in many places, the text communicates an underlying assumption that the LFPs studied are locally generated. This is apparent, for instance, from sentences like, “Some evidence suggests that gamma emerges from fast-spiking interneurons (FSIs) within the striatum...” (line 58-59) and “...to investigate striatal gamma” (line 76). In this case, there is a substantial literature on how pyramidal-interneuron (PING) or interneuron-only (ING) microcircuits may generate gamma rhythms (Buzsaki & Wang, 2006; 2012) but these spiking models only translate into a corresponding LFP under certain conditions that may not be met in the striatum (see the point in Linden et al. 2011 on the contribution of stellate cells to their simulated LFP, which are similar in morphology to many striatal neurons).

To some extent, this overall issue can be addressed with clarifications in the text. However, there is also a deeper problem: a crucial component (and strength) of the study is the conversion from the model activity to a simulated LFP. It appears the way this is currently done is with a 3D Gaussian kernel centered on each FSI in the model. However, as shown in Linden et al. Neuron 2011, the spatial distribution of synapses on radially symmetric cells (they simulated a stellate cell, but those are more similar to striatal neurons in morphology compared to a typical pyramidal cell) does not generate a sizable LFP. How do the simulation results in the current study change if a more realistic model of striatal sinks and sources was used instead of the 3D Gaussian kernel?

I agree with Rev 1 about the importance of considering inter-electrode distance in some of the analyses. I agree that a more explicit connection to what these results mean for HD pathophysiology -- perhaps by additional analyses correlating some of their analyses with symptoms, and/or by additional discussion, would strengthen the paper.

Minor comments:

*The authors cite a number of studies examining LFP oscillations in the ventral striatum. I agree that these are relevant, but the authors may want to point out that (to my knowledge, anyway) the few studies that have compared dorsal and ventral striatal LFPs (e.g. Berke et al. Neuron 2004; Berke, EJN 2009) report some differences, such as larger gamma in the ventral areas. Some passages in the text read as if the authors regard all striatal subfields as one and the same (e.g. by freely mixing references to dorsal and ventral areas in line 52-53).

*Line 89, “Both models were symptomatic...”. Given the various correlations that have been reported between behavior and (ventral) striatal LFPs (Howe et al. PNAS 2011; von Nicolai et al. JNsci 2014; Donnelly et al. PLoS ONE 2014; Malhotra et al. EJN 2015) is it possible that symptoms such as reduced motor activity can explain some of the differences observed between Htt and WT mice? Specifically, given increased delta and gamma during rest in rats as compared to moving, do WT mice look like the Htt mice when only behaviorally identical epochs are compared (e.g. rest, running)?

*Some clarifications regarding the data analysis would be useful:

- Lines 357 and following: am I correct that this analysis is done for all possible striatum-cortex pairs? Or only specific selected pairs?

- If the assumption is that cortical delta drives striatal delta oscillations, shouldn't there be a phase lag between the two resulting from the synaptic time constant, rather than the zero phase lag reported?

- Line 432. “Correlations within a structure were higher than between structures...” I don't think this result can be used to argue against volume conduction unless the inter-electrode distances in both groups are comparable. If the average distance between electrodes in striatum and cortex is larger compared to within-area distances, this would be expected to yield lower correlations simply based on distance.

- What is the reason for detecting gamma events to show phase-amplitude coupling rather than simply showing average power at different phases (fewer parameters)?

*Please clarify the logic of using delta-band filtered cortical activity as the synaptic drive into striatum. Why not the unfiltered signal?

*Discussion, line 552: “Thus, motor cortical inputs to a model FSI network alone cannot reproduce the more heterogeneous distribution of gamma locking to delta phases observed in HD mice” -- I thought the WT mice, not the HD mice, had a more heterogenous distribution of gamma power wrt delta phase, typo?

## Author Response

Synthesis Statement for Author (Required):

I and the same two reviewers have discussed your resubmission extensively. Both reviewers are impressed with the work and find it to be very interesting. They are also impressed with the great deal of extra work you put into this resubmission. There are a couple of additional concerns that need to be addressed, however.

The most important concern is that there is considerable uncertainty regarding the treatment of whether the gamma is local or not. Neither reviewer was convinced that that gamma is locally generated. Much of the uncertainty is because you are making interpretations of a model that is somewhat limited, and your experiments were not designed to test those hypotheses. The concerns below do not need new animal experiments, but each should be addressed appropriately. I will list the reviewers concerns below, along with what I expect you to do in response to each one:

1) The updated “random field direction” model of LFP generation is an important improvement, because it reflects more closely the morphology and resulting extracellular fields expected for radially symmetric FSIs. However, this scenario has the property that as more units are added, the sum of the generated fields will tend to approach zero. (This is already visible in the lower overall power in the PSDs in Supplementary Figure 2C compared to A and B.) The authors should comment on what this implies for extrapolations of the model to realistic numbers of neurons and synapses, including MSNs and their synaptic interactions with FSIs.

ED: please add clarify and discuss

Authors: We indeed observe that the overall lower PSD when the LFP is computed from the model using units with random field orientations. Our intent was to convey that although the power is globally decreased, the gamma peak persists.

Regarding the number of units, we also agree that as more units are added (each with a random

orientation), lower PSD results, and in the limit as the number of units goes to infinity, the spectrum will become flat (i.e. linear). We scaled the model to account for a realistic number of neurons and synapses, albeit only for the FSI population. We described in the Methods (section Network structure, ll.366) the population statistics from animal studies from which we derived our scaling target (e.g. Gittis et al. 2010).

We re-emphasized in the paper that a model MSN population will be needed to gain a more

realistic picture of striatal dynamics, and the resulting fields generated will likely be affected (section starting l.789). However, our assumptions expressed in the Introduction maintain that FSIs are the most likely source of gamma. Furthermore, there is to our knowledge no study showing MSN to FSI synaptic interactions, but only FSI-MSN feedforward inhibition (Bennett and Bolam, 1994; Koos and Tepper, 1999; Gittis et al., 2010; Planert et al., 2010). For these reasons, our model focuses on the FSI population in isolation.

Another rigorous study of FSI-MSN interactions will add to the picture in a subsequent publication (e.g. Chartove et al. 2017, SfN Abstracts), but note that to date, even the larger computational models of the basal ganglia circuitry cannot scale to realistic numbers of neurons and synapses (e.g. Lindahl & Hellgren-Kotaleski 2016) to compute the LFP (added ll.799-802).

2) The authors provide a new imaginary coherence analysis, interpreted to argue against the possibility that striatally recorded gamma oscillations in the LFP are volume conducted. However, they should first acknowledge that there is a highly relevant example of gamma being volume conducted several millimeters into the striatum from the piriform cortex (Berke et al. EJN 2009; Carmichael et al. JNSci 2017).

Authors: Done (ll.501-504, l.684).

In addition, it seems that their results (Figure 6B) may well be consistent with volume conduction: certainly in the WT case, the bulk of the data points clusters around 0. This analysis is sensitive to “false positive” gamma events and to contamination of gamma by noise, so a more convincing analysis would need to determine if as gamma event quality increased (higher power, multiple cycles less likely to be due to non-oscillatory transients) the coherency became more, or less, clustered around 0.

ED: can you add an additional analysis as suggested? Does this change the interpretation (see #4 below)?

Authors: In the previous reviewed version, the coherence was computed for all quiet rest periods, chunked in 3 second blocks. Some blocks did not contain gamma events, some

contained many. We agree that this analysis was affected by noise when the power in the gamma band is very low and we therefore updated the computational routine as suggested.

We now compute the coherence between channels during gamma events only (therefore when power is significantly high in at least one of the 2 channels), and it can be noted that the

imaginary parts do not spread as much as in the previous case, reflecting volume-conducted components as mentioned by the reviewer, although it still shows a variety of values spread

along the real and imaginary axes (see Suppl. Figure 3 below and revised Figure 6B). This observation is now reported in the Results section (ll.506-510), and we suggest that

although part of the signal for some channel pairs is likely volume conducted, there remains a significant part of the signal for other channel pairs which is endogenously generated (non-zero

imaginary part and real part between 0 and 1).

3) Ultimately, these experiments are not designed to localize the source(s) of striatal LFPs, and I don't think the authors need to solve that problem for their contributions to be useful. However, the above considerations suggest that locally generated LFP components, such as those simulated by their model network, certainly are only part of the striatally recorded LFP. I think it would increase the impact of the work if the authors explained under what conditions the predictions of their model are best tested/their interpretations are most valid -- for instance, with future LFP recordings/analyses/experimental manipulations that can exclude volume-conducted components and focus on local components only.

ED: Please add the suggested discussion

Authors: One experiment to test our predictions would involve selectively blocking MSN spiking in awake resting WT and HD mice (at symptomatic age if possible). The closest study to our knowledge was published by Klaus & Plenz (PLoS Biol. 2016) and selectively removed GABAergic transmission in striatum or synaptic excitation onto FSIs and MSNs. They studied the consequences of such pharmacological blockade in cortical spiking statistics (avalanches),

striatal spiking correlation (via MUA, the LFP being less significant), and movement initiation. A similar setup, but instead pharmacologically or optogenetically silencing the MSN population (and not FSIs) would match closely the microcircuit modeled in our simulations. Furthermore, in awake animals adding multi-unit recordings would allow interpretation of spiking statistics with respect to cortical slow oscillations, and further disambiguate volume-conduction vs. local microcircuit sources. We added a discussion of this proposed experiment to the discussion

(ll.846-854).

4) The implications of the interpretation being incorrect are important: if it turned out the LFPs they are analyzing and simulating are mostly non-local, that would change the interpretation substantially. They would have discovered something about how septal/piriform/olfactory bulb activity is altered in Htt mutant mice, and their modeling efforts would be better directed at simulating spiking rather than LFP activity.

ED: Address this in the discussion after completing #2.

Authors: As we discussed in answering #2 above, our revised coherence analysis has now been applied to gamma events only (instead of entire quiet rest periods) and suggests that although

part of the striatal signal is likely volume conducted, there remains a substantial part of the signal that is not (non-zero imaginary part and real part between 0 and 1).

We furthermore added the following paragraph in the discussion (section “Relation to pathophysiology and behavioral states”) to explain consequences of the purely volume-

conducted striatal signal scenario, and additional interpretations (ll.735-748).

In case of a purely volume-conducted signal from nearby cortical regions (such as the sensori- motor cortex), the interpretation of our analysis would apply to a cortical-only source of gamma, most likely generated by the interaction of pyramidal cells (PY) and interneurons (IN), specifically via perisomatic inhibition of PY by parvalbumin-positive (PV) IN (Buzsaki & Wang

2012). Intriguingly, the consequences of such an interpretation on behavioral aspects would be highly relevant to HD pathophysiology: sensori-motor gamma oscillations have been related to active walking in humans (Seeber et al. 2015), with distinctive functional processes related to movement initiation and sustained movement in high (75-100 Hz) and low (35-50 Hz) gamma bands (respectively) in a visuo-motor task (Crone et al. 1998). Our data analysis also supports a disruption in cortically generated gamma oscillations (Figure 1, blue traces, and Figure 4, top row), but our computational model architecture does not explicitly account for an excitatory spiking populations interacting with the FSIs.

Hippocampal gamma, on the other hand, is less likely to occur at rest in healthy condition, and its cross-frequency coupling is usually associated with memory recall in goal directed tasks in rodents (Johnson & Redish 2007; Tort et al. 2008; van der Meer et al. 2011), which is not congruent with Huntington's disease symptomatology.

5) One thing I haven't been able to figure out is if the imaginary coherence analysis is confined

to identified gamma episodes. They don't say in the Methods but the figure caption implies that it is (confined to gamma episodes). That would affect the point above about the overly-sensitive gamma event detection algorithm influencing that result.

ED: Please clarify

Authors: As mentioned above the coherence was previously computed for all quiet rest periods (chunked in 3 second blocks, not only during gamma events). We updated the Methods to describe clearly the new computation of coherence during gamma transients in one of the 2 channels (ll. 267) and in Figure 6B legend. We note that whether the coherence is restricted to gamma events or not, the real part of the coherence is higher within structures (Ctx-Ctx, Str-Str) than across structures (Ctx-Str), and the imaginary part follows the inverse trend. This reflects that volume conduction effect is a priori stronger in closely localized electrodes, which is expected.

There were two other concerns about methods from Rev 1. Please respond to both:

6) The authors answered my question regarding grounds and references but did not add that information to the Methods (I don't think). Sorry if I missed it, I think it is important for readers to know what the references and grounds were for the recordings (note that reference and ground are not synonymous and it should be stated explicitly what was used for each).

Authors: This is now explicitly mentioned line 106-108:

“Cortical channels are referenced to cortical reference electrode and striatal channels are

referenced to striatal reference electrode (both reference electrodes are connected to the recording system's amplifier ground)”.

7) Regarding my (Rev 1) original point 4 - that I think their method would extract not only gamma-specific power fluctuations but also any wideband power increase. The appendix clearly shows events that are wide-band power increases that were identified as gamma events (for example, HD 39-08 and WT 45-09, which looks like chewing artifact). This probably does not affect their overall results much (these seem to be rare), but I can't tell for sure how frequently this happens. My guess is the net results would not change if they put in an exclusion criterion that any Wideband signal exceeding a fixed threshold cannot be counted as a gamma event. That would, however, increase confidence in the results.

Authors: We implemented the exclusion criteria by refining the method that extract events in the low gamma frequency band (25-55Hz) to reject events corresponding also to a transient increase in either alpha (8-12Hz), beta (13-24Hz) and high gamma (&gt;60Hz) bands (see ll. 201-203). Figures 4 & 5 have been updated accordingly, and as suspected, exclusion does not change the outcomes of the analysis.
